# Physiological Responses, Cadmium Partitioning, and Mineral Nutrient Disruption in *Brassicaceae* Crops Exposed to Cadmium Stress

**DOI:** 10.3390/plants15071019

**Published:** 2026-03-26

**Authors:** Halil Samet

**Affiliations:** Department of Crop and Animal Production, Izmit Vocational School, Kocaeli University, Kocaeli 41280, Türkiye; halil.samet@kocaeli.edu.tr

**Keywords:** Cadmium stress, *Brassicaceae*, metal partitioning, nutrient imbalance, oxidative stress

## Abstract

Cadmium (Cd) contamination of agricultural soils poses a serious threat to crop productivity and food safety due to its high mobility, bioaccumulation potential, and toxicity. This study investigated the effects of increasing Cd levels on growth performance, physiological responses, Cd partitioning, mineral nutrient disruption, and Cd accumulation in four *Brassicaceae* crops (cress, watercress, broccoli, and white cabbage). Plants were grown in plastic pots filled with 4 kg of soil under controlled greenhouse conditions and exposed to five different Cd concentrations (0, 5, 10, 20, and 50 mg kg^−1^). Cd exposure significantly affected growth and physiological responses in a species-dependent manner. Compared to the control, shoot dry weight decreased by up to 66.4% in broccoli and 51.7% in cress at the highest Cd level, while white cabbage exhibited comparatively greater tolerance. Oxidative stress indicators showed contrasting patterns, with hydrogen peroxide (H_2_O_2_) increasing by up to 8.8-fold, whereas proline and membrane permeability (MP) responses varied among species. Photosynthetic pigments declined in cress but increased in broccoli under high Cd conditions, suggesting differential adaptive strategies. Cd accumulated predominantly in roots; however, root retention capacity declined at elevated Cd concentrations (20–50 mg kg^−1^ soil), leading to greater Cd translocation to shoots. Elevated translocation factors and shoot Cd distribution demonstrated that physiological tolerance did not necessarily limit Cd accumulation in edible tissues. Cd stress also induced notable imbalances in essential mineral nutrients, particularly potassium (K), calcium (Ca), and zinc (Zn), reflecting strong Cd–nutrient interactions at uptake and transport levels. These nutrient disruptions not only exacerbated physiological stress responses but also reduced the nutritional quality of plant tissues. Notably, species maintaining relatively stable growth under moderate Cd exposure still accumulated substantial Cd concentrations in shoots, highlighting a critical disconnect between agronomic performance and food safety. In conclusion, the findings demonstrate that *Brassicaceae* crops exhibit contrasting strategies in response to Cd stress, with significant implications for Cd entry into the food chain. The study emphasizes the importance of integrating physiological assessment with metal partitioning and nutrient balance analyses when evaluating crop suitability for cultivation in Cd-contaminated soils and for mitigating potential risks to human health.

## 1. Introduction

Cadmium (Cd) is a non-essential and highly toxic trace metal that has become a major environmental and food-safety concern due to its persistence in soils and its efficient transfer from soil to plants and subsequently to humans through the food chain [1,2]. Cd contamination of agricultural soils originates from both natural and anthropogenic sources, including parent material weathering, atmospheric deposition, phosphate fertilizers, mining and smelting activities, sewage sludge application, and irrigation with contaminated water [3,4]. Once introduced into soil, Cd can remain bioavailable for extended periods depending on soil physicochemical properties such as pH, organic matter content, carbonate level, and texture, thereby posing a long-term risk to crop production and food safety [5].

Dietary exposure represents the primary route of Cd intake in non-smoking populations, accounting for approximately 90% of total human exposure. In addition, chronic Cd intake is associated with renal tubular dysfunction, skeletal damage, and other systemic effects due to its long biological half-life, which may exceed 20–30 years in the human body [6]. In response to these health risks, international authorities have established intake-based safety thresholds. The European Food Safety Authority (EFSA) defined a tolerable weekly intake (TWI) of 2.5 µg Cd kg^−1^ body weight. In comparison, the Joint FAO/WHO Expert Committee on Food Additives (JECFA) established a provisional tolerable monthly intake (PTMI) of 25 µg Cd kg^−1^ body weight, reflecting Cd’s cumulative toxicity [7,8]. These guidelines emphasize the importance of minimizing Cd accumulation in edible crops, particularly vegetables.

Vegetables constitute a major component of human diets worldwide and are recognized as significant contributors to dietary Cd intake, especially in regions with intensive vegetable consumption or Cd-contaminated soils. Furthermore, leafy vegetables are of particular concern because they are often consumed fresh or minimally processed and may exhibit relatively high Cd accumulation, even under moderate soil contamination [9]. Importantly, visible growth inhibition does not always correlate with Cd concentrations in edible tissues, meaning that crops may appear agronomically tolerant yet still pose a food-safety risk [10].

Cd contamination in agricultural soils has become an increasing global concern due to its persistence, mobility, and potential entry into the food chain. Background Cd concentrations in uncontaminated soils typically range between 0.1 and 0.5 mg kg^−1^, whereas slightly contaminated agricultural soils may contain approximately 1–3 mg kg^−1^ Cd. Moderately polluted soils often exhibit concentrations between 3 and 10 mg kg^−1^, while heavily contaminated soils influenced by industrial activities, mining, or long-term phosphate fertilizer application may exceed 20–50 mg kg^−1^ Cd. Major sources of Cd accumulation include phosphate fertilizers, industrial emissions, wastewater irrigation, and atmospheric deposition [2,4,5]. Therefore, evaluating plant responses across a gradient representing low to highly contaminated conditions is essential for understanding crop performance and food safety risks under realistic environmental scenarios [2]. However, despite extensive research on Cd toxicity, species-specific differences in Cd accumulation, partitioning, and physiological responses remain insufficiently understood, particularly among *Brassicaceae* crops widely consumed as vegetables.

The *Brassicaceae* family includes numerous economically important and widely consumed vegetables such as cabbage, broccoli, cress, and rocket-type greens [11]. Members of this family are also well known for their capacity to take up and translocate heavy metals, a feature that has attracted attention in both phytoremediation and food-safety research [12]. However, *Brassicaceae* crops are not uniform in their responses to Cd exposure. Species-dependent differences have been reported in Cd uptake efficiency, root-to-shoot translocation, and chelation by phytochelatins, vacuolar sequestration, and interactions with essential nutrients such as Ca, K, Zn, and Fe [13,14]. These differences can result in contrasting patterns of Cd accumulation and tolerance among closely related crops.

At the physiological level, Cd interferes with multiple metabolic processes, including nutrient uptake, photosynthesis, and membrane integrity. Although Cd is not a redox-active metal, it induces oxidative stress indirectly by disrupting electron transport chains and weakening antioxidant defense systems, leading to excessive production of reactive oxygen species (ROS) [15,16]. Consequently, Cd stress is commonly associated with reductions in photosynthetic pigment contents, increased MP, lipid peroxidation, and accumulation of stress-related metabolites such as proline [17]. The intensity of these responses generally increases with Cd dose but varies substantially among species and genotypes.

From a food-chain perspective, the internal distribution of Cd between roots and shoots is particularly important in *Brassicaceae* crops because shoot tissues constitute the primary edible organs. Quantitative indices such as the bio-concentration factor (BCF), translocation factor (TF), and total accumulation rate (TAR) are widely used to describe Cd uptake efficiency, internal transport, and whole-plant accumulation dynamics [18,19]. When evaluated together with growth and physiological traits, these indices allow differentiation between crops that restrict Cd translocation to edible tissues and those that may represent a higher dietary risk despite maintaining biomass production.

Despite extensive research on Cd toxicity, comparative studies evaluating multiple *Brassicaceae* crops under identical soil conditions and graded Cd levels remain limited. Many previous studies have focused on single species or hydroponic systems, which may not fully represent soil-based exposure scenarios encountered in agricultural practice. Therefore, soil-based pot experiments incorporating multiple *Brassicaceae* crops provide valuable insights into crop-specific Cd responses under realistic conditions and allow integration of plant performance with food-safety considerations.

Recent studies have highlighted increasing concern regarding Cd contamination in agricultural soils in various regions of Turkey, driven by industrial emissions, long-term fertilizer use, irrigation practices, and atmospheric deposition. Reported Cd concentrations in Turkish agricultural soils vary depending on regional characteristics, ranging from near-background levels in relatively uncontaminated areas to elevated concentrations in industrially influenced or intensively cultivated zones [20,21]. Similar patterns of spatial variability and anthropogenic influence have been reported globally, emphasizing the need to evaluate crop responses across environmentally relevant contamination gradients [2]. Therefore, the Cd concentrations applied in this study (0, 5, 10, 20, and 50 mg kg^−1^ soil) were selected to represent a gradient from background to heavily contaminated agricultural soils, enabling interpretation of species-specific responses under realistic environmental scenarios and strengthening the relevance of the findings for crop management and food safety risk assessment.

Despite extensive studies on Cd toxicity, comparative evaluations integrating physiological responses, Cd partitioning dynamics, and nutrient imbalance across multiple *Brassicaceae* crops under identical soil conditions remain limited, particularly from a food safety perspective.

In this study, we investigated the responses of four *Brassicaceae* crops—white cabbage (*Brassica oleracea* var. *capitata*), broccoli *(Brassica oleracea* var. *italica*), cress (*Lepidium sativum*), and watercress (*Eruca vesicaria*)—to increasing soil Cd levels (0, 5, 10, 20, and 50 mg kg^−1^ soil supplied as CdCl_2_) under greenhouse conditions. The study aimed to (i) quantify species-specific growth and physiological responses to graded Cd stress; (ii) evaluate Cd uptake, accumulation, and partitioning between roots and shoots; and (iii) assess nutrient imbalance and oxidative stress dynamics in relation to potential food safety risks. By integrating physiological traits with Cd distribution patterns, this work seeks to provide comparative insights into tolerance mechanisms and crop suitability under Cd-contaminated conditions.

## 2. Results

### 2.1. Effects of Cadmium on Plant Growth and Biomass Production

#### 2.1.1. Dry Weights (DWs) in the Shoot and Root

Increasing Cd levels significantly affected biomass production (shoot and root DWs) in all *Brassicaceae* crops; however, the magnitude of reduction differed markedly among species. In general, low Cd levels (5–10 mg kg^−1^) caused limited or no visible reduction in fresh and dry biomass, whereas higher Cd concentrations (particularly 50 mg kg^−1^) resulted in pronounced growth inhibition (Table 1).

In cress, the shoot DW decreased progressively with Cd exposure, showing reductions of 15.3–26.6% at 5–20 mg kg^−1^ Cd and a pronounced decline at 50 mg kg^−1^ Cd (51.7%) relative to the control. In watercress, the shoot DW was not significantly affected at low Cd levels (5–10 mg kg^−1^), but a significant decrease was found at higher Cd concentrations (20 and 50 mg kg^−1^) by 17.2% and 37.6%, respectively. Broccoli exhibited the strongest growth inhibition, with the shoot DW decreasing continuously from 5 mg kg^−1^ Cd onward and reaching a 66.4% reduction at 50 mg kg^−1^ Cd. In contrast, white cabbage maintained stable shoot DW at low and moderate Cd levels (5–20 mg kg^−1^), while a significant decrease (43.2%) was observed only at 50 mg kg^−1^ Cd (Table 1).

In cress, the root DW remained relatively stable at low Cd levels (5 and 10 mg kg^−1^ Cd), but declined sharply at higher Cd concentrations. Compared with the control, root DW decreased by 41.1% at 20 mg kg^−1^ Cd and reached the lowest value at 50 mg kg^−1^ Cd, with a 74.4% reduction, indicating severe inhibition of root growth under high Cd stress. In watercress and broccoli, root DW showed a gradual but statistically nonsignificant decline with increasing Cd exposure. Relative to the control, reductions in watercress ranged from 13.9% to 28.5% at 5 to 50 mg kg^−1^ Cd. In addition, the reductions in root DW in broccoli decreased with Cd levels (5, 10, 20, and 50 mg kg^−1^) by 31.3%, 50.0%, 54.2%, and 72.9%, respectively. In white cabbage, root DW remained stable at low and moderate Cd levels (5–20 mg kg^−1^), while a significant reduction was observed at 50 mg kg^−1^ Cd, corresponding to a 51.4% decrease compared with the control (Table 1).

#### 2.1.2. Dry Matter Contents (DMCs) in the Shoot and Root

Changes in shoot and root DMC reflected Cd-induced alterations in biomass accumulation and tissue hydration status across *Brassicaceae* crops. The shoot DMC was significantly affected by increasing Cd levels in test plants (Table 1). In cress, the shoot DMC remained relatively stable at low Cd levels (5 and 10 mg kg^−1^), but declined markedly at higher Cd concentrations. Compared with the control, the shoot DMC decreased by 31.3% at 20 mg kg^−1^ Cd and reached the lowest value at 50 mg kg^−1^ Cd with a 52.1% reduction, indicating a strong negative effect of Cd on dry matter accumulation. In watercress, the shoot DMC showed a gradual decline with increasing Cd exposure. Reductions ranged from 11.9% at 5 mg kg^−1^ Cd to 30.9% at 50 mg kg^−1^ Cd. Broccoli exhibited a pronounced reduction in the shoot DMC at moderate and high Cd levels. Relative to the control, the shoot DMC decreased by 40.9% and 37.7% at 10 and 20 mg kg^−1^ Cd, respectively, while a partial recovery was observed at 50 mg kg^−1^ Cd, but values remained significantly lower than the control. In white cabbage, the shoot DMC was not significantly affected by Cd exposure at low and moderate levels (5–20 mg kg^−1^). However, a significant decline was observed at the highest Cd level (50 mg kg^−1^), with the shoot DMC decreasing by 27.3% compared with the control (Table 1).

In cress, the root DMC showed a slight increase at 5 mg kg^−1^ (4.9%) but declined at higher Cd concentrations. Compared with the control, the root DMC decreased by 18.5% at 10 mg kg^−1^ Cd and reached the lowest value at 50 mg kg^−1^ Cd, corresponding to a 57.4% reduction, indicating strong impairment of dry matter accumulation in roots under severe Cd stress. In watercress, root DMC decreased progressively with increasing Cd exposure. Reductions ranged from 11.0% at 5 mg kg^−1^ Cd to 52.0% at 50 mg kg^−1^ Cd. Broccoli exhibited a pronounced reduction in root DMC across all Cd treatments. Relative to the control, it declined sharply at 5 mg kg^−1^ Cd (66.9%) and remained markedly lower at 10, 20, and 50 mg kg^−1^ Cd. In white cabbage, the root DMC was not significantly affected by Cd exposure. However, the root DMC tended to increase at 20 and 50 mg kg^−1^ Cd levels by 13.1% and 48.9%, respectively, in comparison with the control (Table 1).

#### 2.1.3. Cadmium Distribution in the Shoots and Roots

This section focuses on the relative distribution of Cd between shoot and root tissues, expressed as percentage partitioning within the plant.

When shoot and root Cd distribution data are evaluated together, a clear inverse relationship between Cd allocation to shoots and roots becomes evident across all *Brassicaceae* crops (Table 1). In cress, the shoot Cd distribution increased sharply at 5, 10, and 20 mg kg^−1^ Cd levels by 4.6-, 4.4-, and 3-fold, respectively, more than the control, while root Cd distribution significantly declined to approximately 47.0, 45.4, and 26.0%, respectively. Notable effects of the highest Cd level on Cd distribution in the shoot and root were not found. The lower Cd levels caused an increase in the shoot Cd distribution, whereas higher Cd levels increased the root Cd distribution. In watercress, changes in the shoot Cd distribution were relatively limited, and the root Cd distribution remained consistently high across Cd treatments. Broccoli displayed strong root-dominant Cd retention at all Cd levels. The shoot Cd distribution remained very low at low and moderate Cd treatments, corresponding to the root Cd distribution values exceeding 96%. A notable exception occurred at Cd50, where a marked increase in shoot Cd distribution (6.6-fold) coincided with a decrease in root Cd distribution, indicating that extremely high Cd exposure can partially overcome root retention capacity. In white cabbage, the shoot Cd distribution increased markedly at all Cd levels, particularly at 5 mg kg^−1^ Cd, where the shoot Cd distribution increased nearly sixfold, while the root Cd distribution declined to its lowest value. At higher Cd levels, the shoot Cd distribution remained elevated, although the root Cd distribution gradually increased again (Table 1).

### 2.2. Cadmium Concentration, Accumulation, and Translocation

In contrast to the distribution analysis presented above, this section describes the absolute Cd concentrations in plant tissues and derived accumulation indices, including bio-concentration factor (BCF), translocation factor (TF), and total accumulation rate (TAR).

Cd concentration, accumulation, and translocation were significantly affected by increasing Cd levels in all *Brassicaceae* crops, with pronounced species-dependent differences observed in both the shoot and root tissues (Table 2).

#### 2.2.1. Cadmium Concentration in the Shoots and Roots

The Shoot Cd concentration increased markedly with increasing Cd levels in all crops (Table 2). In cress, the shoot Cd rose sharply from 0.80 mg kg^−1^ in the control to 29.60 mg kg^−1^ at 5 mg kg^−1^ Cd and increased progressively, reaching 172.80 mg kg^−1^ at 50 mg kg^−1^ Cd, corresponding to a 216-fold increase relative to the control. In watercress, the shoot Cd increased from 1.20 mg kg^−1^ in the control group to 195.2 mg kg^−1^ at the highest Cd level (50 mg kg^−1^), representing a 163-fold increase. In broccoli, the shoot Cd concentration showed a consistent dose-dependent increase, rising from 0.93 mg kg^−1^ (control) to 172.3 mg kg^−1^ (50 mg kg^−1^) by a 185-fold increase. The highest shoot Cd accumulation was observed in white cabbage, where the shoot Cd increased from 0.60 mg kg^−1^ in the control group to 251.6 mg kg^−1^ at 50 mg kg^−1^ Cd, corresponding to a 419-fold increase compared with the control.

The root Cd concentrations were also increased sharply with increasing Cd levels in all genera (Table 2). In cress, the root Cd increased progressively from 6.10 mg kg^−1^ in the control to 26.10 mg kg^−1^ at 5 mg kg^−1^ Cd and 51.40 mg kg^−1^ at 10 mg kg^−1^ Cd. A pronounced increase was observed at higher Cd levels, with root Cd reaching 159.90 mg kg^−1^ at 20 mg kg^−1^ Cd and 1063.10 mg kg^−1^ at 50 mg kg^−1^ Cd, corresponding to a 174-fold increase relative to the control. In watercress, the root Cd exhibited a steep and continuous increase across Cd treatments. Compared with the control (7.30 mg kg^−1^), the root Cd increased to 127.90 mg kg^−1^ at 5 mg kg^−1^ Cd and reached 1500.10 mg kg^−1^ at 50 mg kg^−1^ Cd, representing a 205-fold increase. DMRT analysis indicated significant differences among all Cd treatments. Broccoli showed relatively high basal root Cd levels (48.70 mg kg^−1^) and a consistent dose-dependent increase under Cd exposure. Root Cd concentration increased to 249.00 mg kg^−1^ at 5 and 386.10 mg kg^−1^ at 10 mg kg^−1^ Cd, reaching 1229.30 mg kg^−1^ at 50 mg kg^−1^ Cd, corresponding to a 25-fold increase compared with the control. In white cabbage, the root Cd increased markedly with increasing Cd levels. Relative to the control (18.20 mg kg^−1^), the root Cd rose to 118.90 mg kg^−1^ at 5 mg kg^−1^ Cd and reached 1462.90 mg kg^−1^ and 50 mg kg^−1^ Cd, representing an 80-fold increase.

#### 2.2.2. Bio-Concentration Factors (BCF) of the Shoots and Roots

Table 2 indicates that the BCF values in the shoots and roots of *Brassicaceae* crops were significantly influenced by increasing Cd levels, with clear and consistent species-dependent trends observed across all varieties.

The shoot BCF showed a marked decline with increasing Cd application in all crops. In cress, the shoot BCF decreased sharply from 20.0 in the control to 5.9–5.5 at 5–10 mg kg^−1^ Cd and further to 3.4 at 50 mg kg^−1^ Cd, corresponding to a reduction of more than 80% relative to the control. A similar pattern was observed in watercress, where shoot BCF declined from 30.0 in the control to 3.9 at a 50 mg kg^−1^ Cd level. In broccoli, the shoot BCF exhibited the strongest suppression under Cd exposure. Compared with the control (23.3), the shoot BCF values dropped drastically at all Cd treatments, reaching 1.2–1.4 at 5–20 mg kg^−1^ Cd levels and remaining very low at 50 mg kg^−1^ Cd (3.4). In white cabbage, the shoot BCF declined from 15.0 in the control to 3.2–5.3 across Cd treatments, indicating a consistent reduction in the shoot Cd accumulation efficiency with increasing Cd levels (Table 2).

The root BCF values were consistently much higher than the shoot BCF values across all crops and Cd treatments. However, the root BCF also decreased markedly with increasing Cd concentration. In cress, the root BCF declined from 153.3 in the control to 5.1–8.0 at 5–20 mg kg^−1^ Cd, followed by a partial increase to 21.3 at 50 mg kg^−1^ Cd. In watercress, the root BCF decreased from 183.3 in the control to approximately 25–33 across all Cd treatments. Broccoli exhibited extremely high root BCF values under control conditions (1217.2), which declined sharply under Cd exposure, reaching 49.4 at 5 mg kg^−1^ Cd and falling to 4.6 at 50 mg kg^−1^ Cd. Similarly, white cabbage showed a strong reduction in the root BCF from 455.0 in the control to 23.6–29.2 under Cd treatments.

#### 2.2.3. Translocation Factors (TFs)

Increasing Cd levels significantly influenced the percentage of TF, with pronounced species- and dose-dependent differences observed among *Brassicaceae* crops (Table 2).

In cress, TF increased sharply at low and moderate Cd levels. Compared with the control (13.33), TF increased to 114.10 at 5 mg kg^−1^ Cd and 107.83 at 10 mg kg^−1^ Cd, corresponding to more than an eightfold increase. Although TF declined at higher Cd concentrations, values at 20 mg kg^−1^ Cd (53.13) remained substantially higher than the control, while 50 mg kg^−1^ Cd showed TF values comparable to the control. In watercress, TF values remained relatively stable across all Cd treatments. Minor increases were observed at 5 and 10 mg kg^−1^ Cd levels, whereas slight reductions occurred at 20 and 50 mg kg^−1^ Cd values. Broccoli exhibited a progressive increase in TF with increasing Cd exposure. Relative to the control (1.93), TF increased gradually from 2.93 at 5 mg kg^−1^ to 4.41 at 20 mg kg^−1^ Cd and showed a marked increase at 50 mg kg^−1^ Cd, reaching 14.07, corresponding to a 7.3-fold increase. In white cabbage, TF increased significantly at all Cd treatments. The highest TF value was observed at 5 mg kg^−1^ Cd (22.50, a 6.8-fold increase), while elevated TF values were also recorded at 10, 20, and 50 mg kg^−1^ Cd levels, indicating enhanced Cd translocation from roots to shoots under Cd exposure.

#### 2.2.4. Total Accumulation Rate (TAR)

The values of TAR increased significantly with increasing Cd levels in all *Brassicaceae* crops, although the magnitude of increase varied considerably among species (Table 2).

In cress, TAR increased progressively from 1.83 in the control to 17.40 at 5 mg kg^−1^ Cd and 31.67 at 10 mg kg^−1^ Cd. Further increases were observed at higher Cd levels, with TAR reaching 42.03 at 20 mg kg^−1^ and 47.87 at 50 mg kg^−1^ Cd, corresponding to a 26-fold increase relative to the control. In watercress, TAR exhibited a strong and continuous increase across Cd treatments. Compared with the control (1.80), TAR increased to 34.33 at 5 mg kg^−1^ Cd and 97.00 at 10 mg kg^−1^ Cd, reaching the highest value at 50 mg kg^−1^ Cd (166.03), which represented a 92-fold increase relative to the control. Broccoli showed comparatively lower TAR values than other crops. TAR increased from 0.37 in the control to 3.93–3.97 at 5 and 10 mg kg^−1^ Cd and reached 7.10 at 20 mg kg^−1^ Cd, while a slight decrease was observed at 50 mg kg^−1^ Cd. In white cabbage, TAR increased sharply with increasing Cd exposure. TAR rose from 2.13 in the control to 26.60 at 5 mg kg^−1^ Cd and 55.57 at 10 mg kg^−1^ Cd, reaching 75.30 and 88.60 at 20 and 50 mg kg^−1^ Cd levels, respectively, corresponding to a 42-fold increase compared with the control.

### 2.3. Effects of Cadmium on Photosynthetic Pigments

**Figure 1:**
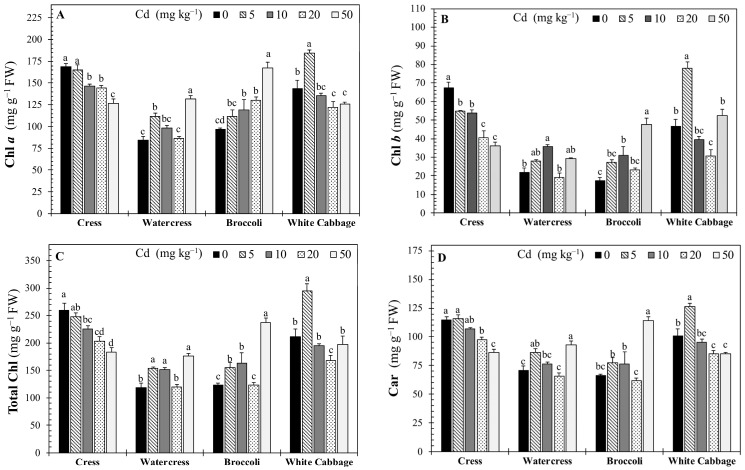
The effects of Cd on (**A**) chlorophyll *a*, (**B**) chlorophyll *b*, (**C**) total chlorophyll, and (**D**) carotenoid content of some *Brassica* crops. Values are means ± SE (*n* = 3). Different letters indicate significant differences according to DMRT (*p* < 0.05).

Cd exposure significantly affected the contents of photosynthetic pigments in all crops (Figure 1). Increasing Cd levels resulted in a progressive decline in Chl *a*, Chl *b*, total Chl, and Car contents, although the severity of the reduction differed among species.

In cress, Chl *a* content decreased progressively with increasing Cd concentration. While 5 mg kg^−1^ Cd caused nonsignificant change compared to the control, Chl *a* declined significantly at the 10 and 20 mg kg^−1^ Cd levels, reaching its lowest value at the 50 mg kg^−1^ Cd level, corresponding to a 24.8% reduction relative to the control (Figure 1A). In watercress, Chl *a* content increased significantly at low and high Cd levels. Compared with the control, Chl *a* increased by 33.2% at the 5 mg kg^−1^ Cd level and reached the maximum at the 50 mg kg^−1^ Cd level, showing a 56.4% increase. Broccoli exhibited a dose-dependent stimulatory response of Chl *a* to Cd exposure. Chl *a* content increased significantly from the 5 mg kg^−1^ Cd level onward and reached the highest value at the 50 mg kg^−1^ Cd level, showing a 72.6% increase compared with the control. In white cabbage, Chl *a* content increased significantly at the 5 mg kg^−1^ Cd level (28.6%) but declined at higher Cd levels. Chl *a* values at the 20 mg kg^−1^ and the 50 mg kg^−1^ Cd levels were significantly lower than the control (15.0% and 12.4% decrease, respectively) (Figure 1A).

In cress, compared to the control, Chl *b* declined significantly by 19.0% and 20.4% at the 5 and 10 mg kg^−1^ Cd levels (Figure 1B). Notable decreases were observed with higher Cd levels, reaching 40.0% and 46.4% at the 20 and 50 mg kg^−1^ Cd levels, respectively. In watercress, Chl *b* content increased significantly at the 5 and 10 mg kg^−1^ Cd levels, showing increases of 27.6% and 63.1%, respectively, compared with the control. However, Chl *b* declined at the 20 mg kg^−1^ Cd level and partially recovered at the 50 mg kg^−1^ Cd level (34.2%). Broccoli exhibited a strong stimulatory response of Chl *b* to Cd exposure. Chl *b* content increased markedly at the 5 and 10 mg kg^−1^ Cd levels and reached the highest value at the 50 mg kg^−1^ Cd level, corresponding to a 2.7-fold increase relative to the control. In white cabbage, Chl *b* content increased significantly at the 5 mg kg^−1^ Cd level (67.2%), but declined at the 10 and 20 mg kg^−1^ Cd levels, and showed partial recovery at the 50 mg kg^−1^ Cd level (Figure 1B).

In cress, total Chl content decreased progressively with increasing Cd concentration. (Figure 1C) Compared with the control, total Chl declined by 4.5% at 5 mg kg^−1^ Cd, 13.0% at 10 mg kg^−1^ Cd, and 21.6% at 20 mg kg^−1^ Cd, and reached the lowest value at 50 mg kg^−1^ Cd, with a 29.4% reduction, indicating a consistent inhibitory effect of Cd on total chlorophyll accumulation. In watercress, total Chl content increased significantly at the 5 and 10 mg kg^−1^ Cd levels, showing increases of 29.7% and 27.7%, respectively, relative to the control. While no marked change was observed at 5 mg kg^−1^ Cd, total Chl reached the highest value at 50 mg kg^−1^ Cd, corresponding to a 48.4% increase compared with the control. Broccoli exhibited a pronounced stimulatory response of total Chl to Cd exposure. total Chl content increased significantly at 5 and 10 mg kg^−1^ Cd, remained comparable to the control at 20 mg kg^−1^ Cd, and increased sharply at 50 mg kg^−1^ Cd, reaching a 1.9-fold increase relative to the control. In white cabbage, total Chl content increased significantly at Cd5 (39.7%) but declined at higher Cd concentrations. Significant reductions were observed at 10 and 20 mg kg^−1^ Cd, whereas the total Chl content at 50 mg kg^−1^ was slightly below the control (Figure 1C).

In cress, Car content remained unchanged at 5 mg kg^−1^ Cd but declined progressively at higher Cd concentrations (Figure 1D). Compared with the control, it decreased by 6.9% at 10 mg kg^−1^ Cd, 15.2% at 20 mg kg^−1^ Cd, and reached the lowest value at 50 mg kg^−1^ Cd, with a 25.1% reduction. In watercress, Car content increased significantly at 5 mg kg^−1^ Cd (21.8%) and reached the highest value at 50 mg kg^−1^ Cd, corresponding to a 31.1% increase relative to the control. In contrast, a slight reduction was observed at 20 mg kg^−1^ Cd. Broccoli exhibited a biphasic response of Car content to Cd stress. Car levels increased at 5 and 10 mg kg^−1^ Cd levels, declined slightly at 20 mg kg^−1^ Cd, and increased sharply at 50 mg kg^−1^ Cd, reaching a 1.7-fold increase compared with the control. In white cabbage, Car content showed a strong stimulatory response to Cd exposure. A dramatic increase was observed at 5 mg kg^−1^ Cd, with Car content increasing by 4.9-fold relative to the control. Although Car levels declined at higher Cd concentrations, values at 10, 20, and 50 mg kg^−1^ Cd levels remained more than 3-fold higher than the control (Figure 1D).

### 2.4. Oxidative Stress Indicators

**Figure 2:**
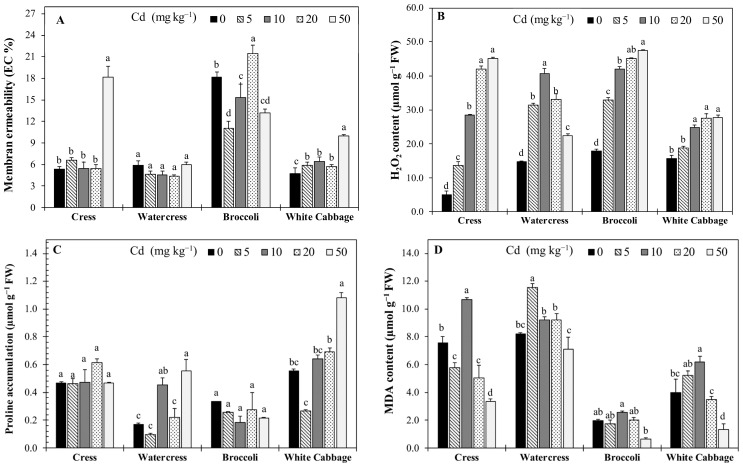
The effects of Cd on (**A**) membrane permeability (MP), (**B**) H_2_O_2_ content, (**C**) proline accumulation, and (**D**) MDA content of some *Brassica* crops. Values are means ± SE (*n* = 3). Different letters indicate significant differences according to DMRT (*p* < 0.05).

Oxidative stress parameters responded sensitively to Cd exposure in all *Brassicaceae* crops (Figure 2). The MP percentage responded sensitively to increasing Cd levels, with pronounced species-dependent differences (Figure 2A). In cress, MP increased dramatically at 50 mg kg^−1^ Cd, reaching a 3.4-fold rise compared with the control, indicating severe membrane damage. In contrast, watercress maintained stable MP values across all Cd treatments, with no significant differences among Cd levels. Broccoli exhibited a biphasic response, showing reduced MP at low Cd levels followed by a significant increase at 20 mg kg^−1^ Cd. White cabbage displayed a progressive increase in MP with rising Cd concentrations, with a more than twofold increase at the highest Cd level.

The H_2_O_2_ content increased significantly with increasing Cd levels in all *Brassicaceae* crops, although the magnitude and pattern of response varied markedly among species (Figure 2B). In cress, H_2_O_2_ concentration showed a strong and progressive increase with Cd dose. Compared with the control, H_2_O_2_ levels increased with Cd levels (5, 10, 20, and 50 mg kg^−1^) by 2.6-, 5.5- 8.2-, and 8.8-fold more, respectively, indicating a severe oxidative response under high Cd exposure. In watercress, H_2_O_2_ content increased significantly at low and moderate Cd levels, reaching a maximum at 10 mg kg^−1^ with a 2.8-fold increase compared to the control. However, H_2_O_2_ levels declined at higher Cd concentrations (20 and 50 mg kg^−1^), although remaining significantly above control values, suggesting a non-linear response pattern. Broccoli exhibited a gradual and dose-dependent increase in H_2_O_2_ accumulation across all Cd treatments. In comparison with the control, H_2_O_2_ content increased by 1.8-fold at 5 mg kg^−1^ and reached approximately a 2.7-fold increase at 50 mg kg^−1^. In white cabbage, H_2_O_2_ accumulation increased moderately but consistently with increasing Cd concentration. Significant increases were observed from Cd10 onward, with H_2_O_2_ levels reaching approximately 1.8-fold of the control at 20 and 50 mg kg^−1^.

Proline accumulation responded differentially to increasing Cd levels among *Brassicaceae* crops, showing clear species-dependent patterns (Figure 2C). In cress, proline accumulations remained relatively stable across Cd treatments. Compared with the control, only a moderate increase (29.8%) was observed at the 20 mg kg^−1^ Cd level, while no significant changes were detected at the other Cd levels. In watercress, proline accumulation showed a pronounced and dose-dependent response. Proline content decreased significantly at 5 mg kg^−1^ (41% reduction relative to the control), increased markedly at 10 mg kg^−1^ (2.6-fold), and reached the highest value at 50 mg kg^−1^, corresponding to a 3.3-fold increase compared with the control. These changes were statistically significant, indicating strong induction of proline accumulation at higher Cd levels. Broccoli exhibited no significant induction of proline accumulation under Cd stress. Proline content decreased relative to the control across all Cd treatments, with reductions ranging from 18% to 45%; however, no statistically significant differences among treatments were detected according to DMRT. In white cabbage, proline accumulation increased progressively with increasing Cd concentration. After an initial decrease at the 5 mg kg^−1^ Cd level (53% reduction), proline levels increased at the 10 and 20 mg kg^−1^ Cd levels and reached a maximum at the 50 mg kg^−1^ Cd level, showing an approximately twofold increase compared with the control. These changes were statistically significant and indicated a clear Cd dose-dependent response.

The MDA content, an indicator of lipid peroxidation, exhibited significant and species-dependent changes in response to increasing Cd levels (Figure 2D). In cress, MDA content increased significantly at the 10 mg kg^−1^ Cd level, showing a 41% increase compared to the control, whereas lower (5 mg kg^−1^) and higher Cd levels (20 and 50 mg kg^−1^ Cd levels) resulted in significantly reduced MDA values compared with the control. The lowest MDA content in cress was observed at the 50 mg kg^−1^ Cd level, corresponding to a 56% reduction compared to the control. In watercress, MDA content increased significantly at the 5 mg kg^−1^ Cd level, reaching a 1.4-fold increase compared with the control. Moderate increases were also observed at the other Cd levels, while MDA content declined at the 50 mg kg^−1^ Cd level, falling below the control level. Broccoli exhibited relatively low MDA levels across all Cd treatments. A significant increase was observed only at the 10 mg kg^−1^ Cd level (30% increase), whereas MDA content decreased at the 50 mg kg^−1^ Cd level, showing a marked 68% reduction compared to the control. Overall, differences among Cd treatments in broccoli were less pronounced than in other crops. In white cabbage, MDA content increased significantly at the 5 and 10 mg kg^−1^ Cd levels, with the highest value recorded at the 10 mg kg^−1^ Cd level (with a 55% increase compared with the control). However, MDA levels declined sharply at higher Cd concentrations, with reductions of 13% at Cd20 and 66% at Cd50, indicating decreased lipid peroxidation at the highest Cd exposure.

### 2.5. Effects of Cadmium on Mineral Ion Concentrations

#### 2.5.1. Potassium Concentrations in Shoots and Roots

The shoot and root K concentrations were significantly affected by increasing Cd levels, with species-specific response patterns observed across crops (Table 3).

In cress, the shoot K concentrations increased progressively with increasing Cd levels. Compared with the control, the shoot K increased slightly at 5 and 10 mg kg^−1^ Cd, whereas a pronounced increase was observed at 20 and 50 mg kg^−1^ Cd, by 53.8% and 82.0%, respectively. In watercress, the shoot K concentrations showed moderate fluctuations in response to Cd exposure. Minor increases were observed at 5, 20, and 50 mg kg^−1^ Cd levels. Broccoli exhibited a contrasting pattern, with the shoot K content increasing significantly at 5 mg kg^−1^ Cd (17.9%) and 10 mg kg^−1^ Cd (9.4%), followed by a decline at higher Cd levels (21.5) compared with the control. In white cabbage, the shoot K concentrations remained relatively stable across Cd treatments, showing only minor increases. The highest shoot K value was observed at 50 mg kg^−1^ Cd (10.9% increase), while no marked reductions were detected at any Cd level.

The root K concentration in cress declined significantly at 5 mg kg^−1^ Cd (29.0%), but gradually recovered at higher Cd levels; the root K concentration in the 50 mg kg^−1^ Cd application exceeded the control value by 15.3%, indicating partial restoration of the root K levels under severe Cd stress. In watercress, root K concentration increased markedly at 5 and 10 mg kg^−1^ Cd levels by 31.8% and 47.5%, respectively. The highest root K value was observed at 20 mg kg^−1^ Cd (57.8% increase), followed by a slight decline at the 50 mg kg^−1^ Cd level. Broccoli showed a strong and progressive decline in the root K concentration with increasing Cd levels. Relative to the control, the root K decreased by 11.6% at 5 mg kg^−1^ Cd, 42.2% at 10 mg kg^−1^ Cd, and reached the lowest value at 50 mg kg^−1^ Cd, showing a 66.9% reduction. In white cabbage, the root K concentration remained relatively stable at low Cd levels, while a significant increase was observed at 20 mg kg^−1^ Cd (24.0%). In contrast, 50 mg kg^−1^ Cd caused a notable decrease in the root K concentration by 29.8% compared with the control.

#### 2.5.2. Calcium Concentrations in Shoots and Roots

The shoot and root Ca concentration was significantly affected by increasing Cd levels, with clear species-dependent response patterns observed among *Brassicaceae* crops (Table 3).

In cress, the shoot Ca content showed minor and non-significant reductions at 5 and 10 mg kg^−1^ Cd levels, followed by an increase at the 20 mg kg^−1^ Cd level. A pronounced increase was observed at 50 mg kg^−1^ Cd, where the shoot Ca content reached 5.00, corresponding to a 6.0-fold increase relative to the control. In watercress, the shoot Ca concentration showed non-significant increases with increasing Cd concentration except for the 50 mg kg^−1^ Cd level. The shoot Cd concentration increased considerably with this Cd level by 69.5% compared with the control. The effect of applied Cd levels on the shoot Ca concentration of broccoli was non-significant, and it exhibited relatively stable values across all Cd treatments. In white cabbage, the shoot Ca concentrations remained largely unchanged at low and moderate Cd levels but increased significantly at 50 mg kg^−1^ Cd, corresponding to a 32.3% increase compared with the control.

The root Ca concentrations in cress showed slight fluctuations at low and moderate Cd levels but increased markedly at 50 mg kg^−1^ Cd, reaching 0.48 mg kg^−1^, which represented an 84.6% increase relative to the control. In watercress, the root Ca concentration tended to decrease at the lowest Cd level but tended to increase at higher Cd levels. Broccoli showed a contrasting response, with the root Ca increasing substantially at 5 and 10 mg kg^−1^ Cd (1.6–1.7-fold) but declining sharply at higher Cd concentrations. At 20 and 50 mg kg^−1^ Cd levels, the root Ca decreased by more than 50% relative to the control. In white cabbage, the root Ca content exhibited a non-significant decrease/increase with all Cd levels, but at the highest Cd level, the root Ca concentration reached 2.89 mg kg^−1^, corresponding to a 4.6-fold considerable increase compared with the control (Table 3).

#### 2.5.3. Zinc Concentrations in Shoots and Roots

The shoot and root Zn concentrations responded strongly to Cd exposure, with consistent increases observed across all *Brassicaceae* crops, particularly at higher Cd levels (Table 3).

In cress, the shoot Zn concentration increased significantly at 10 and 20 mg kg^−1^ Cd, reaching the highest value at 20 mg kg^−1^ Cd (44.5% increase relative to the control). At 50 mg kg^−1^ Cd, the shoot Zn declined and returned to a level comparable to the control. In watercress, the shoot Zn concentration increased progressively with increasing Cd concentration. Compared with the control, this parameter increased significantly at 10, 20, and 50 mg kg^−1^ Cd levels by 24.4%, 26.5%, and 64.3%, respectively. Broccoli exhibited a non-linear response of the shoot Zn to Cd exposure. While a notable decrease in the shoot Zn concentration was observed at the 10 mg kg^−1^ Cd level, it increased markedly at higher Cd levels, with increases of 92.1% at 20 mg kg^−1^ Cd and 149.4% at 50 mg kg^−1^ Cd compared with the control. In white cabbage, the shoot Zn concentration decreased substantially at 5, 10, and 20 mg kg^−1^ Cd, showing reductions of approximately 33–37% relative to the control. At 50 mg kg^−1^ Cd, the shoot Zn partially recovered but remained slightly below the control value.

The root Zn concentrations in cress showed a marked increase at 20 mg kg^−1^ Cd (2.4-fold) and reached the highest value at 50 mg kg^−1^ Cd, corresponding to a 3.2-fold increase relative to the control. In watercress, the root Zn concentration increased substantially in all Cd treatments. Relative to the control, the root Zn increased with 5 and 10 mg kg^−1^ Cd levels by 72.3% and 79.1%, respectively. In higher Cd levels (20 and 50 mg kg^−1^), the shoot Zn concentration increased remarkably by 84.4 and 141.5 mg kg^−1^, respectively. Broccoli showed relatively moderate changes in the root Zn concentration. While the root Zn declined at 10 mg kg^−1^ Cd, it increased at 50 mg kg^−1^ Cd, with the highest value recorded at this Cd level (23.7%). In white cabbage, the root content increased markedly at higher Cd concentrations. Compared with the control, the root Zn increased by 79.5% and 73.6% at 20 and 50 mg kg^−1^ Cd levels, respectively, indicating enhanced Zn accumulation in roots under elevated Cd exposure (Table 3).

### 2.6. Correlation Matrix Analyses

**Figure 3:**
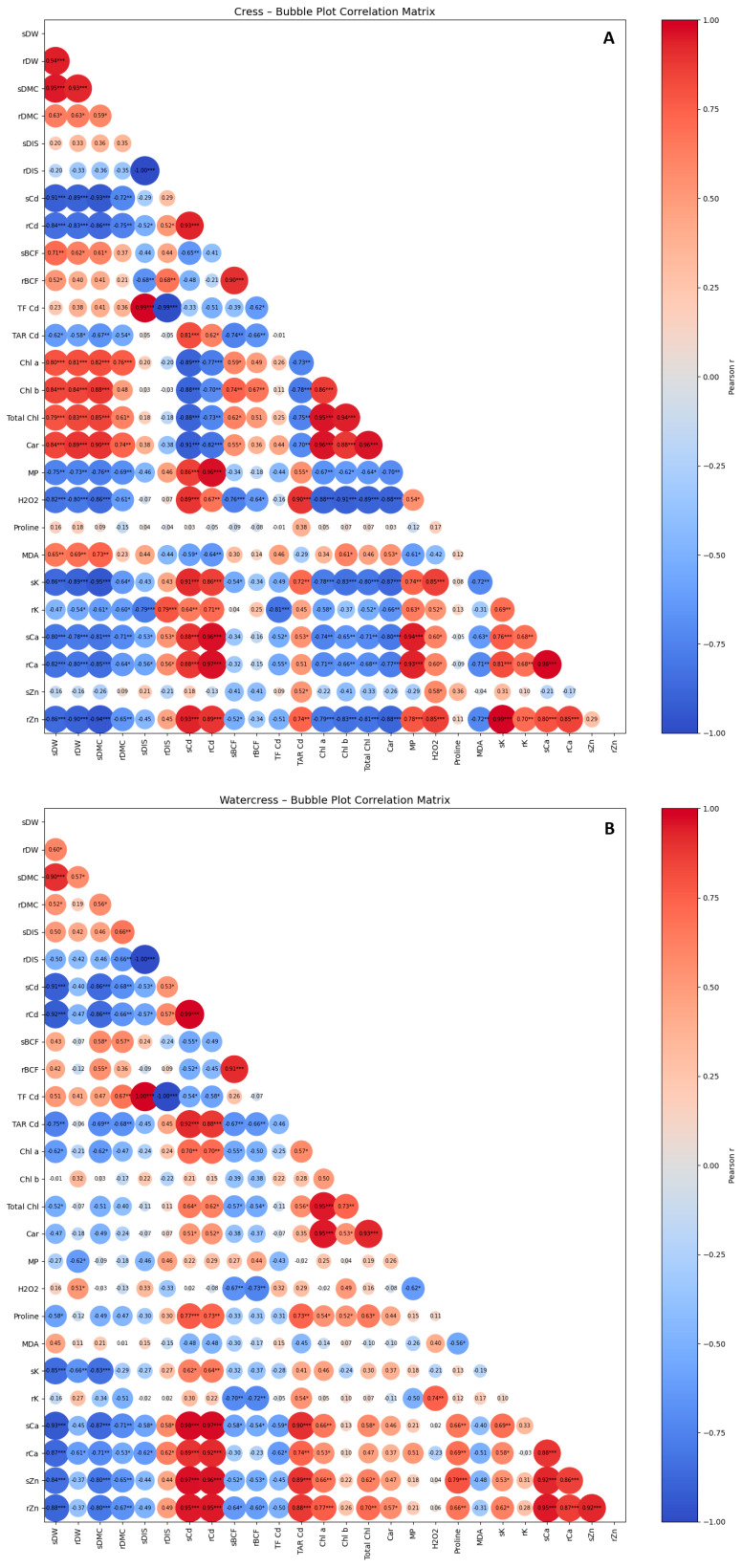

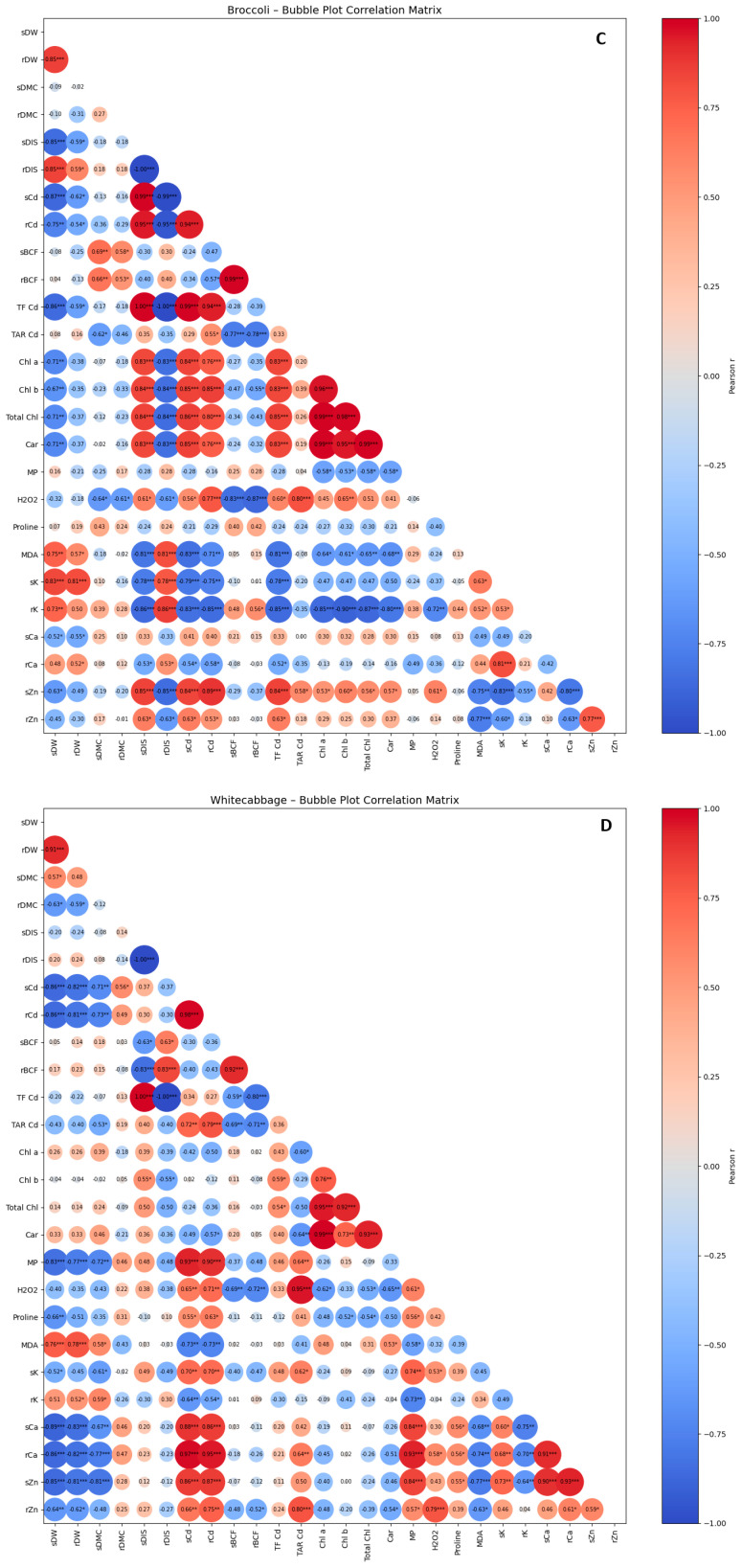
Correlation matrices illustrating the relationships among physiological, biochemical, and cadmium-related parameters measured in the roots and shoots of (**A**) cress, (**B**) watercress, (**C**) broccoli, and (**D**) white cabbage. Circle color and size represent the direction and strength of Pearson correlation coefficients, respectively: red circles indicate positive correlations, whereas blue circles indicate negative correlations. Larger circles correspond to stronger correlation coefficients. Asterisks denote levels of statistical significance (*: *p* < 0.05, **: *p* < 0.01, ***: *p* < 0.001). Abbreviations: r, root; s, shoot; DW, dry weight; DMC, dry matter content; Cd, cadmium; DIS, Cd distribution index; BCF, bio-concentration factor; TF, translocation factor; TAR, total accumulation ratio; Chl, chlorophyll; Car, carotenoid; MP, membrane permeability; H_2_O_2_, hydrogen peroxide; MDA, malondialdehyde.

Correlation matrix analyses were performed to elucidate the relationships among growth traits, photosynthetic pigments, oxidative stress indicators, mineral nutrient contents, and Cd accumulation and translocation parameters across the studied *Brassicaceae* species (Figure 3).

In cress, the strongest positive correlations were observed between (i) shoot and root Cd concentrations (r ≈ 0.93), (ii) Cd concentration and Cd accumulation indices (BCF, TF, TAR; r ≈ 0.85–0.95), and (iii) Cd concentration and oxidative stress markers (H_2_O_2_, MDA; r > 0.85). In contrast, the strongest negative correlations occurred between (i) Cd concentration and biomass traits (sDW, rDW; r < −0.85), (ii) Cd-related parameters and photosynthetic pigments (r ≈ −0.75 to −0.90), and (iii) Cd concentration and potassium content (r ≈ −0.80). These relationships indicate a strong coupling between Cd accumulation, oxidative stress, and growth inhibition in cress (Figure 3A).

In watercress, the most pronounced positive correlations were found between (i) shoot and root Cd concentrations (r ≈ 0.99), (ii) Cd concentration and Zn content in shoots and roots (r ≈ 0.70–0.85), and (iii) proline content and oxidative stress markers (r ≈ 0.75). The strongest negative correlations involved (i) Cd-related parameters and potassium content (r ≈ −0.65 to −0.80), (ii) oxidative stress markers and photosynthetic pigments (r ≈ −0.60 to −0.75), and (iii) Cd concentration and growth traits (r ≈ −0.60). Overall, these moderate correlations suggest a buffered physiological response to Cd stress in watercress (Figure 3B).

In broccoli, the strongest positive correlations were observed between (i) Cd concentration and oxidative stress markers (H_2_O_2_, MDA, MP; r > 0.80), (ii) Cd concentration and translocation factor (r ≈ 0.85), and (iii) oxidative stress markers and pigment degradation (r ≈ 0.80–0.90). Conversely, the strongest negative correlations occurred between (i) Cd concentration and biomass traits (r < −0.80), (ii) Cd-related parameters and photosynthetic pigments (r ≈ −0.85), and (iii) Cd concentration and potassium content (r ≈ −0.75). These patterns indicate limited buffering capacity, with Cd uptake rapidly translated into oxidative damage and growth suppression (Figure 3C).

In white cabbage, the strongest positive correlations were found between (i) shoot and root Cd concentrations (r ≈ 0.98), (ii) Cd translocation indices (TF, TAR) and shoot Cd (r ≈ 0.80–0.90), and (iii) oxidative stress markers and calcium content (r ≈ 0.75–0.85). The strongest negative correlations were observed between (i) Cd concentration and biomass traits (r ≈ −0.70 to −0.80), (ii) Cd-related parameters and photosynthetic pigments (r ≈ −0.65 to −0.80), and (iii) Cd concentration and potassium content (r ≈ −0.75). This pattern reflects partial maintenance of growth despite strong Cd translocation to shoots (Figure 3D).

## 3. Discussion

The severity of Cd-induced damage varies widely among plant species and is influenced by differences in uptake efficiency, internal distribution, detoxification capacity, and stress response mechanisms. Importantly, Cd tolerance does not necessarily correlate with reduced Cd accumulation in edible tissues, emphasizing the need for integrated evaluation of physiological responses together with metal partitioning when assessing crop performance and food safety risks under Cd stress [2]. The Cd concentrations applied in this study represent environmentally relevant contamination levels ranging from background conditions to heavily polluted agricultural soils, allowing species-specific responses to be interpreted within realistic soil pollution scenarios. Within this framework, the following sections discuss the effects of Cd exposure on growth performance, physiological adjustments, metal uptake and translocation dynamics, and nutrient homeostasis in *Brassicaceae* crops. Members of this family are of particular interest in Cd-related research due to their widespread consumption, high nutritional value, and, in some cases, their strong capacity to absorb and accumulate heavy metals. Several *Brassicaceae* species have been reported to exhibit contrasting strategies in response to Cd stress, ranging from effective root retention and detoxification to enhanced root-to-shoot translocation [14,22]. This diversity makes Brassicaceae crops suitable model systems for investigating Cd-induced toxicity, tolerance mechanisms, and associated risks to human health. These improvements were implemented to provide a more mechanistic interpretation of Cd-induced physiological responses and to enhance the clarity of the experimental design and analytical procedures.

These responses are likely governed by interconnected regulatory mechanisms involving transporter-mediated metal uptake, redox imbalance, and stress signaling pathways rather than a single toxicity factor [2,13,14]. Taken together, the coordinated patterns observed among Cd accumulation, oxidative stress responses, nutrient imbalance, and growth inhibition suggest that species-specific tolerance is governed not by a single trait but by the integrated regulation of metal uptake, internal partitioning, and physiological adjustment mechanisms, as widely reported in Cd stress studies [2,13,22].

### 3.1. Cadmium-Induced Growth Inhibition and Biomass Allocation

Cd exposure caused pronounced growth inhibition and altered biomass allocation patterns in all *Brassicaceae* crops examined, confirming Cd as one of the most phytotoxic non-essential metals affecting plant development even at relatively low concentrations (Table 1). The reduction in biomass observed under increasing Cd levels was more pronounced in broccoli compared to other species. This pronounced decline in shoot biomass suggests a reduced capacity to maintain metabolic stability under Cd-induced oxidative and ionic stress, likely reflecting weaker detoxification or ion homeostasis mechanisms.

The observed reductions in the shoot and root DW and DMC with increasing Cd levels are consistent with numerous studies reporting growth suppression as a primary response to Cd stress [23,24].

Growth inhibition under Cd stress has been attributed to multiple, often interacting mechanisms, including inhibition of cell division and elongation, disruption of photosynthetic carbon assimilation, and impairment of nutrient and water uptake [15]. In our study, the most severe biomass losses occurred at the highest Cd level (50 mg kg^−1^), particularly in cress and broccoli, where both shoot and root biomass declined sharply (Table 1). Such sensitivity suggests limited capacity for Cd exclusion or detoxification in these species, leading to early onset of growth penalties [25].

Roots exhibited pronounced sensitivity to Cd exposure, especially at higher Cd levels, which could be related to their direct contact with excess Cd. Accumulation of Cd in the roots can damage root meristems, disrupt membrane integrity, and impair root elongation, thereby limiting water and nutrient acquisition [26]. The strong reduction in the root DW observed in cress and broccoli at increasing Cd concentrations likely contributed to secondary limitations in shoot growth, reflecting the tight coupling between belowground function and aboveground biomass production (Table 1). Although Cd is not redox-active, the disruption of electron transport chains and indirect ROS generation may impair metabolic efficiency and limit carbon assimilation, contributing to biomass reduction [15,16].

In contrast, white cabbage maintained relatively stable shoot biomass and dry matter content at low and moderate Cd levels, indicating a higher degree of tolerance. Such tolerance may be associated with more effective Cd sequestration in roots, enhanced binding of Cd to cell wall components, or compartmentalization within vacuoles, thereby reducing Cd interference with metabolic processes in actively growing tissues [13,27]. Watercress similarly exhibited moderate resilience at lower Cd doses, although growth inhibition became evident at higher Cd exposure (Table 1).

The shoot and root DMC percentage provided additional insight into Cd-induced shifts in biomass allocation (Table 1). The decline in DMC under high Cd stress suggests impaired structural carbon accumulation and altered tissue hydration status, a response commonly associated with stress-induced metabolic reprogramming [24,28]. Reduced DMC reflects a prioritization of maintenance and stress defense over growth, as resources are diverted toward detoxification, antioxidant defense, and cellular repair processes [29]. The pronounced interspecific variation observed in growth and biomass responses highlights the importance of species-specific Cd tolerance strategies within the *Brassicaceae* family. Differences in growth sensitivity among crops likely arise from variation in Cd uptake rates, root-to-shoot translocation efficiency, and internal detoxification capacity, as well as differential regulation of hormonal and metabolic pathways controlling growth [14,24].

Overall, the present findings demonstrate that Cd stress severely constrains biomass production and alters biomass allocation in *Brassicaceae* crops, with severity dependent on both Cd concentration and plant species (Table 1). These growth responses not only have direct implications for crop productivity but also provide a physiological context for understanding Cd accumulation patterns and their potential consequences for food quality and human health, which are discussed in subsequent sections.

### 3.2. Photosynthetic Pigments and Oxidative Stress Responses

Cd exposure significantly altered photosynthetic pigment contents and induced oxidative stress in all *Brassicaceae* crops investigated, consistent with numerous reports demonstrating that Cd disrupts photosynthetic apparatus and redox homeostasis in higher plants [15,24]. In the present study, Chl *a* and *b*, total Chl, and Car contents exhibited distinct species-dependent modulation under increasing Cd levels (Figure 1), and these changes were closely associated with multiple oxidative stress indicators, including MP, H_2_O_2_, MDA, and proline accumulation (Figure 2).

Chl *a* and *b* are central to light harvesting and energy conversion in the thylakoid membrane, and their depletion under heavy metal stress has been widely documented [30]. In cress, both Chl *a* and *b* contents declined progressively with increasing Cd levels, with reductions reaching approximately 25% and 46%, respectively, at the highest Cd concentration compared to the control (Figure 1A,B). These decreases indicate impairment of pigment biosynthesis or accelerated degradation. Such reductions are likely linked to Cd-induced disruption of chlorophyll biosynthetic enzymes and the degradation of chloroplast ultrastructure [23]. Similarly, total Chl and Car contents decreased by approximately 30% and 25% in cress at 50 mg kg^−1^ Cd, reflecting compromised photosynthetic capacity consistent with the substantial growth inhibition observed in this species (Figure 1C,D).

By contrast, watercress and broccoli exhibited non-linear or biphasic pigment responses. Watercress showed increases in chlorophyll a and total chlorophyll at certain Cd levels, and broccoli exhibited elevated pigment contents at moderate Cd doses. These apparent stimulatory effects under stress may represent transient adaptive responses, including upregulation of chlorophyll biosynthesis or alterations in leaf morphology that temporarily enhance chlorophyll concentration per unit leaf area [31]. However, increased pigment levels should not be interpreted unambiguously as improved physiological performance; rather, they may reflect stress-induced reallocation of resources or failure to degrade chlorophyll despite oxidative damage. Such patterns have been reported in other heavy metal exposures, where pigment increases were accompanied by oxidative biomarkers, indicating stress rather than tolerance mechanisms [32]. Notably, the magnitude and direction of pigment changes corresponded closely with biomass responses, particularly in cress, where marked chlorophyll depletion paralleled severe growth inhibition, supporting a functional link between photosynthetic impairment and reduced productivity under Cd stress.

Carotenoids, which function as accessory pigments and photoprotective antioxidants, also exhibited species-specific responses (Figure 1D). In white cabbage, Car content increased sharply at moderate Cd levels and remained elevated, a pattern consistent with activation of photoprotective mechanisms to quench excess excitation energy and scavenge singlet oxygen [33]. In cress and watercress, Car contents declined under high Cd levels, possibly due to Cd-induced inhibition of the methylerythritol phosphate pathway or oxidative degradation of Car molecules [34].

The observed pigment dynamics were closely paralleled by markers of oxidative stress. The MP increased significantly with Cd exposure, particularly in cress and white cabbage, indicating increased lipid bilayer disruption. Increased MP is a reliable indicator of loss of membrane integrity under oxidative assault, and has been widely used to quantify heavy metal–induced stress [35]. Similarly, H_2_O_2_ and MDA contents, representing ROS accumulation and lipid peroxidation, respectively, increased with Cd dose in most species (Figure 2B,D). Elevated levels of H_2_O_2_ can act as both a damaging oxidant and a signalling molecule, triggering stress response pathways, including antioxidant enzyme induction [17]. However, under the highest Cd exposure, a contrasting response pattern was observed, where H_2_O_2_ levels remained elevated while MDA content declined. The contrasting responses observed between H_2_O_2_ accumulation and MDA levels under high Cd exposure suggest a complex oxidative balance, where enhanced ROS production may be partially counteracted by protective mechanisms such as osmolyte accumulation or membrane stabilization processes. This indicates that, despite increased ROS generation, lipid peroxidation may be partially controlled under severe stress conditions.

Proline accumulation, a common metabolic response to abiotic stress, was also Cd-responsive in a species-dependent manner. In watercress and white cabbage, proline levels increased markedly with Cd, suggesting activation of osmoprotective and ROS-scavenging mechanisms [36]. In contrast, cress and broccoli exhibited limited proline induction, potentially reflecting differences in the efficiency of proline biosynthesis or partitioning under heavy metal stress (Figure 2C).

The strong negative correlations between Cd-related parameters and biomass traits, together with inverse relationships between Cd accumulation and photosynthetic pigments, indicate that growth inhibition and pigment degradation are tightly linked responses to Cd stress rather than independent processes (Figure 3). Similar correlations have been reported in several Cd-exposed crops, where oxidative damage to chloroplast membranes and inhibition of chlorophyll biosynthesis resulted in reduced photosynthetic efficiency and biomass production [17]. The strong positive associations observed between oxidative stress markers and pigment loss further support the central role of ROS-mediated damage in Cd-induced photosynthetic impairment.

Interestingly, under the highest Cd treatment (50 mg kg^−1^), MDA levels decreased despite elevated H_2_O_2_ accumulation, indicating a non-linear oxidative stress response. Such non-linear responses suggest differential regulation of antioxidant signaling pathways rather than simple accumulation of oxidative damage. Although increased ROS production typically promotes lipid peroxidation, reduced MDA levels under severe stress conditions have been reported in several plant species and may reflect activation of protective mechanisms rather than reduced oxidative pressure. High Cd exposure can induce metabolic adjustments such as enhanced antioxidant enzyme activity, membrane lipid remodeling, or selective degradation of peroxidized lipids, leading to stabilization of membrane integrity despite elevated ROS levels. Additionally, severe stress may suppress growth and metabolic activity, thereby limiting substrate availability for lipid peroxidation processes. Similar decoupling between ROS accumulation and MDA levels has been reported in Cd-stressed plants, where oxidative signaling and damage responses are differentially regulated depending on stress intensity and species-specific tolerance strategies [17,37,38]. Cd-induced ROS accumulation may destabilize thylakoid membranes and interfere with pigment biosynthesis enzymes, explaining the observed species-dependent pigment responses [15,17].

Differences in oxidative damage among species despite comparable or elevated Cd accumulation may reflect variation in antioxidant defense capacity. Cd-induced oxidative stress is commonly mitigated through enzymatic ROS-scavenging systems, including superoxide dismutase (SOD), catalase (CAT), and various peroxidases, which collectively regulate cellular redox balance. Species exhibiting lower levels of lipid peroxidation and membrane damage may possess more efficient antioxidant responses that limit ROS propagation, thereby preserving cellular integrity even under high Cd exposure. Although antioxidant enzyme activities were not directly measured in the present study, the observed physiological patterns are consistent with previous reports demonstrating that enhanced antioxidant capacity contributes significantly to Cd tolerance by reducing oxidative damage rather than preventing Cd uptake itself [17,37,38].

Taken together, these results indicate that Cd exposure triggered pronounced oxidative stress in *Brassicaceae* crops, disrupting photosynthetic pigment stability and triggering both common and species-specific protective responses (Figure 1 and Figure 2). The interplay between pigment modulation and oxidative stress aligns with established models of heavy metal phytotoxicity, where Cd interferes with electron transport chains in chloroplasts and mitochondria, amplifying ROS production and impairing photosynthesis [39]. Importantly, the diversity of responses among species highlights that pigment dynamics cannot be generalized across taxa but rather arise from distinct physiological adjustments governed by species-specific tolerance mechanisms.

Cd exposure induced significant alterations in both photosynthetic pigments and essential mineral nutrients, indicating a tightly interconnected disruption of metabolic processes. In addition, Cd-induced nutrient imbalance, particularly involving K^+^, Ca^2+^, and Zn^2+^, may further compromise photosynthetic performance by affecting stomatal regulation, membrane stability, and the activity of metal-dependent enzymes [40,41]. The disruption of essential nutrients such as K, Ca, and Zn further indicates competitive interactions at uptake and transport levels, which may exacerbate Cd-induced physiological stress and contribute to altered metabolic functioning. These imbalances are likely associated with ionic competition at root uptake sites and disruption of membrane transport systems, ultimately contributing to reduced growth and physiological performance under Cd stress.

Beyond these nutrient-related effects, the strong association between Cd accumulation, oxidative stress markers, and pigment degradation suggests coordinated metabolic disturbances across multiple physiological pathways. Cd exposure is known to impair photosynthetic machinery both directly and indirectly by inducing excessive production of reactive oxygen species (ROS), which can damage chloroplast membranes, disrupt thylakoid ultrastructure, and inhibit chlorophyll biosynthesis [15,17]. The elevated H_2_O_2_ levels observed in several species indicate increased oxidative pressure, which may accelerate lipid peroxidation and promote chlorophyll degradation through membrane destabilization and enzyme inactivation [14,37].

Species-specific differences observed among the *Brassicaceae* crops likely reflect variation in detoxification strategies, including differential ROS scavenging capacity, ion compartmentalization, and transporter-mediated metal sequestration [26]. Collectively, these integrated responses indicate that pigment loss under Cd stress represents a systemic metabolic adjustment rather than a solely direct toxic effect.

### 3.3. Cadmium Uptake, Distribution, and Translocation Mechanisms

Cd uptake and its subsequent distribution between roots and shoots constitute key determinants of both plant tolerance and food safety risk. In the present study, Cd concentration increased markedly in both root and shoot tissues with increasing Cd levels across all Brassicaceae crops, confirming that Cd is readily absorbed by plant roots and efficiently transported within the plant system. However, pronounced species-specific differences were observed in Cd partitioning, bioconcentration, and translocation indices, reflecting distinct physiological strategies for coping with Cd stress (Table 1). Cd uptake and redistribution are likely mediated by broad-spectrum divalent metal transporters, including ZIP and NRAMP families, which facilitate Cd entry via pathways originally evolved for essential nutrients [2,42].

Cd accumulation patterns differed markedly among species, with roots generally acting as the primary sink at lower Cd levels but showing reduced retention capacity under higher Cd exposure. This shift resulted in increased translocation of Cd to shoots in some species, indicating a breakdown in root-based restriction mechanisms. Species-specific differences observed in Cd partitioning and tolerance may be attributed to variations in root sequestration capacity, vacuolar compartmentalization, and metal transport regulation among *Brassicaceae* crops. These mechanisms likely play a critical role in determining both tolerance capacity and Cd accumulation in edible tissues.

Roots consistently accumulated substantially more Cd than shoots, reflecting their function as the primary barrier limiting Cd movement to aerial tissues. Cd entering the root is often immobilized through binding to cell wall components, complexation with organic acids, or sequestration into vacuoles, thereby restricting its translocation [13]. The high root Cd concentrations and distribution percentages observed in this study are consistent with this well-established mechanism [10,26].

In addition to physiological and biochemical factors, species-specific differences in Cd accumulation and tolerance may also be influenced by root architectural traits that determine the extent and spatial pattern of metal uptake. Root length, surface area, and root hair development can significantly affect absorptive capacity and soil–root interactions. Although root morphometric traits were not directly assessed in this study, the observed interspecific variation suggests that structural adaptations may contribute to differential Cd uptake and partitioning and warrant further investigation [22,26].

Despite this general root-dominant accumulation pattern, shoot Cd concentration increased sharply with increasing Cd supply in all crops, demonstrating that root retention mechanisms can be progressively overwhelmed under high Cd exposure (Table 1). This was particularly evident in cress and white cabbage, where shoot Cd distribution and TF values increased markedly at low to moderate Cd levels. Elevated TF values indicate enhanced root-to-shoot Cd transport, which may be mediated by shared transport pathways with essential divalent cations such as Ca^2+^, Zn^2+^, and Fe^2+^ [24,27].

TF analysis further highlighted interspecific differences in Cd mobility. Cress and white cabbage exhibited high TF values at low Cd doses, suggesting limited restriction of Cd movement to shoots during early exposure. In contrast, watercress maintained relatively stable TF values across Cd treatments, indicating tighter regulation of Cd translocation. Broccoli displayed low TF values at low and moderate Cd levels but showed a sharp increase at the highest Cd dose, suggesting that extreme Cd exposure can exceed the capacity of root-based exclusion mechanisms [42]. This pattern suggests that root detoxification capacity may become saturated under high Cd exposure, resulting in enhanced shoot translocation.

Differences in root retention and shoot translocation observed among species may also reflect variation in intracellular Cd compartmentation strategies. Cd tolerance in plants is frequently associated with sequestration mechanisms that limit cytosolic Cd toxicity, including vacuolar storage mediated by tonoplast transporters, binding to cell wall components, and chelation with thiol-rich ligands followed by intracellular detoxification. Enhanced root retention may indicate efficient immobilization through cell wall binding or vacuolar sequestration, thereby restricting Cd mobility, whereas increased shoot translocation could reflect more active xylem loading or reduced sequestration capacity in root tissues. Although subcellular localization analyses were not performed in the present study, the physiological patterns observed are consistent with compartmentation-based tolerance mechanisms widely reported in Cd-exposed plants [13,26,27]. Future investigations using imaging techniques such as synchrotron-based X-ray fluorescence mapping or histochemical localization would provide valuable insight into tissue-specific Cd storage patterns.

The marked decline in shoot and root BCF with increasing Cd concentration reflects a dilution effect and saturation of uptake systems at high external Cd levels. Such decreases in BCF values under elevated Cd supply are commonly reported and indicate that Cd uptake efficiency does not increase linearly with soil Cd concentration [14,43]. In contrast, the TAR value of Cd increased strongly with Cd dose in most crops, demonstrating that whole-plant Cd accumulation continued to rise despite reduced uptake efficiency (Table 1).

The inverse relationship observed between shoot and root Cd distribution further emphasizes the dynamic regulation of Cd partitioning within plants. When shoot Cd distribution increased, a corresponding decrease in root Cd distribution was generally observed, indicating redistribution rather than independent accumulation (Table 1). This trade-off reflects coordinated control of Cd transport processes, likely involving metal transporter families such as ZIP, NRAMP, and HMA proteins, which have been implicated in Cd uptake and long-distance transport [2,43].

The correlation analyses provide strong evidence that Cd toxicity in *Brassicaceae* crops is driven by the close coupling between Cd uptake, internal translocation, and oxidative stress (Figure 3). Strong positive correlations between shoot and root Cd concentrations and oxidative stress markers (H_2_O_2_ and MDA) indicate that enhanced Cd mobility amplifies ROS production, leading to cellular damage. This relationship is consistent with previous studies demonstrating that Cd-induced ROS generation intensifies as Cd is redistributed from roots to shoots, thereby disrupting membrane integrity and metabolic processes [14,15].

Collectively, these findings demonstrate that *Brassicaceae* crops employ diverse strategies to regulate Cd uptake and internal distribution. Species with strong root Cd retention and low TF values may limit Cd transfer to edible shoots, but may still experience root growth inhibition. Conversely, species exhibiting higher Cd translocation to shoots accumulate greater Cd concentrations in aerial tissues, which has important implications for food safety. Thus, physiological tolerance to Cd stress could not necessarily correspond to reduced Cd accumulation in edible plant parts.

### 3.4. Nutrient Imbalance and Implications for Food Safety and Human Health

In this study, K, Ca, and Zn were specifically examined because of their fundamental physiological roles in plant metabolism and their well-documented interactions with Cd during uptake, transport, and cellular homeostasis. These elements represent three distinct but complementary nutrient categories macronutrient (K), secondary macronutrient and signaling ion (Ca), and essential micronutrient (Zn), each of which is known to be highly sensitive to Cd-induced disruption [10,38]. These nutrient alterations likely arise from competitive interactions at membrane transport systems combined with Cd-induced disruption of membrane potential and ion homeostasis [38].

Cd exposure not only disrupts plant growth and physiology but also profoundly alters the uptake, distribution, and homeostasis of essential mineral nutrients. In the present study, increasing Cd levels significantly affected the contents of K, Ca, and Zn in both shoot and root tissues, highlighting nutrient imbalance as a central component of Cd-induced toxicity in *Brassicaceae* crops. Such imbalances have direct implications for crop nutritional quality and human health, particularly given the dietary importance of vegetables in this family.

Potassium, a key macronutrient involved in osmotic regulation, enzyme activation, and stomatal function, exhibited species- and organ-specific responses to Cd stress. While moderate increases in shoot K content were observed in some species at higher Cd levels, root K content frequently declined, particularly in broccoli and white cabbage (Table 3). Cd-induced disruption of K uptake has been attributed to competition at plasma membrane transport sites and damage to root membrane integrity, leading to reduced K^+^ influx and altered cellular ion balance [38]. Although elevated shoot K under stress may reflect compensatory redistribution, such changes do not necessarily indicate improved physiological status and may instead signal ionic imbalance.

Cd toxicity is generally associated with membrane depolarization, inhibition or alteration of K^+^ channel activity, and oxidative damage affecting ion transport processes [15,38]. In the present study, species-dependent responses were evident, with increased K accumulation in most species at the highest Cd level, suggesting adaptive regulation to maintain osmotic balance. In contrast, the pronounced decline in broccoli roots indicates failure to maintain K^+^ homeostasis under excessive Cd stress [22]. Mechanistically, Cd may impair K^+^ uptake by inhibiting plasma membrane H^+^-ATPase activity, modifying channel function, and enhancing K^+^ efflux due to oxidative membrane damage, collectively contributing to disturbed K^+^ homeostasis under Cd exposure [38,43,44,45].

Collectively, these findings suggest that disruption of K^+^ homeostasis under Cd stress represents not only a direct consequence of metal toxicity but also an integrative regulatory response that involves adjustments in ion transport and stress signaling, highlighting the central role of potassium balance in determining species-specific tolerance to Cd exposure.

On the other hand, Ca homeostasis was also markedly affected by Cd exposure (Table 3). Ca plays a dual role as a structural component of cell walls and membranes and as a secondary messenger in stress signalling. The pronounced accumulation of Ca in shoot tissues at high Cd levels, particularly in cress and watercress, may reflect activation of Ca-mediated signalling pathways in response to Cd stress [45]. However, Cd is known to interfere with Ca channels and transporters due to similar ionic radii, thereby disturbing Ca^2+^ signalling and membrane stability [46]. Alterations in root Ca content further suggest impaired uptake and translocation dynamics under Cd stress, which may exacerbate root damage and reduce overall plant resilience. Changes in Ca distribution may also reflect activation of Ca-dependent stress-signaling pathways [47].

In the presented study, Zn, an essential micronutrient involved in enzyme function and antioxidant defense, displayed complex interactions with Cd (Table 3). In several species, Cd exposure enhanced Zn accumulation in roots and, in some cases, shoots. This response likely reflects shared uptake pathways between Cd^2+^ and Zn^2+^, particularly via ZIP and NRAMP transporters [2,27]. While increased Zn accumulation may partially mitigate oxidative damage by supporting antioxidant enzyme activity, excessive Cd-induced Zn redistribution can disrupt Zn homeostasis and does not prevent Cd entry into edible tissues. Moreover, competition between Cd and Zn at uptake and binding sites can impair Zn bioavailability, reducing the nutritional value of crops. In addition, alterations in Zn availability may affect antioxidant enzyme activities and redox balance, while excessive Cd accumulation can further exacerbate Zn deficiency in plant tissues [1].

The nutrient imbalance observed under Cd exposure may arise from multiple interacting mechanisms involving both transporter-level competition and stress-induced physiological disruption. Cd can interfere with potassium uptake by altering membrane potential and inhibiting plasma membrane transport systems, thereby reducing K^+^ acquisition efficiency. Similarly, chemical similarity between Cd^2+^ and essential divalent cations such as Zn^2+^ may result in competition for shared transport pathways, including ZIP-family transporters. In addition, Cd-induced oxidative stress and root metabolic impairment may indirectly affect nutrient uptake capacity by damaging membrane integrity and disrupting energy-dependent transport processes. These combined mechanisms likely contribute to the differential nutrient profiles observed among species, suggesting that nutrient imbalance reflects both direct transporter competition and broader physiological stress responses [13,42].

Correlation patterns involving mineral nutrients highlight nutrient imbalance as a core component of Cd toxicity (Figure 3). The consistent negative correlations between Cd concentration and K content support earlier findings that Cd interferes with K^+^ uptake and retention by inhibiting plasma membrane H^+^-ATPase activity, altering membrane potential, and activating K^+^ efflux channels [44]. In contrast, Ca showed positive correlations with Cd accumulation and oxidative stress indicators, suggesting a compensatory role of Ca^2+^ in membrane stabilization and stress signalling under Cd stress. Ca has been shown to play a structural role in cell walls and membranes and to act as an intracellular signal coordinating responses to environmental and stress cues in plants [46]. At the same time, exogenous Ca has been reported to alleviate Cd-induced oxidative damage and modulate stress-signalling pathways [47].

The strong positive correlations between Cd and Zn concentrations in both shoots and roots further indicate shared uptake and transport pathways, as Cd^2+^ can utilize Zn transporters due to chemical similarity. This interaction has been widely documented in *Brassicaceae* species and other plants exposed to Cd-contaminated environments [13,26].

From a food safety perspective, the combined effects of elevated Cd accumulation and altered nutrient composition are particularly concerning. *Brassicaceae* vegetables are widely consumed for their high mineral and antioxidant content; however, the substantial increase in shoot Cd concentration and translocation observed in this study indicates that edible tissues may accumulate Cd to levels that pose health risks. Chronic dietary intake of Cd has been associated with kidney dysfunction, bone demineralization, and increased risk of cardiovascular and metabolic disorders in humans [6,10].

Comparative evaluation of correlation patterns revealed distinct Cd-handling strategies among the studied species (Figure 3). Strong correlations between Cd accumulation, oxidative stress, and growth inhibition in cress and broccoli indicate high sensitivity to Cd exposure, consistent with earlier reports on Cd-sensitive *Brassica* genotypes [48]. In contrast, watercress exhibited weaker correlations, suggesting partial physiological buffering that limits immediate damage despite Cd uptake.

Notably, white cabbage showed strong correlations between Cd translocation indices and shoot Cd concentration while maintaining relatively stable growth, indicating that physiological tolerance does not necessarily restrict Cd transfer to edible tissues. Similar observations have been reported in leafy vegetables, where efficient Cd translocation increases potential dietary exposure despite limited visible toxicity symptoms [49]. These findings underscore the importance of considering Cd partitioning and nutrient interactions when evaluating food safety risks in *Brassicaceae* crops grown under Cd-contaminated conditions (Figure 3).

Importantly, species exhibiting relatively stable growth or pigment content under Cd stress did not necessarily restrict Cd accumulation in shoots, underscoring that apparent physiological tolerance does not equate to food safety. This decoupling between plant performance and Cd accumulation highlights the need to evaluate both agronomic traits and metal partitioning when assessing crop suitability for cultivation in Cd-contaminated soils.

Species-specific differences in Cd accumulation and translocation observed in this study may also be interpreted in the context of transporter-mediated metal uptake and intracellular handling mechanisms. Cd uptake in plants is largely facilitated by broad-spectrum divalent metal transporters, including members of the ZIP (ZRT/IRT-like Protein) and NRAMP (Natural Resistance-Associated Macrophage Protein) families, which primarily mediate Zn^2+^ and Fe^2+^ transport but can inadvertently transport Cd^2+^ due to chemical similarity. Furthermore, heavy metal ATPases (HMAs) are known to play key roles in xylem loading and root-to-shoot translocation, potentially explaining the strong association between Cd accumulation and translocation factors observed in certain species. In addition, ATP-binding cassette (ABC) transporters contribute to intracellular sequestration and detoxification by facilitating vacuolar compartmentalization of Cd complexes. Variation in the regulation or activity of these transporter systems may underlie the contrasting Cd-handling strategies identified among species, where higher translocation efficiency or stronger root retention likely reflects differential transporter expression or activity. Although gene expression analyses were beyond the scope of the present study, the observed physiological patterns are consistent with transporter-mediated mechanisms widely reported in *Brassicaceae* and other plant species exposed to Cd stress

Notably, the strong correlations observed between Cd accumulation, translocation indices, oxidative stress markers, and nutrient imbalance may indirectly reflect differential regulation or activity of metal transporter systems (e.g., ZIP, NRAMP, HMA, and ABC transporters), suggesting that species-specific physiological responses identified in this study are likely underpinned by transporter-mediated control of Cd uptake, redistribution, and intracellular sequestration [10,13,43].

Collectively, the present findings support an integrative physiological framework in which Cd toxicity is governed by the interaction between metal uptake, redox imbalance, and disruption of mineral nutrient homeostasis. Increased Cd accumulation and internal mobility were closely associated with elevated oxidative stress, suggesting that enhanced Cd translocation amplifies ROS generation, leading to membrane destabilization, pigment degradation, and growth inhibition. The consistent disruption of potassium homeostasis highlights the role of altered ion transport in amplifying Cd toxicity, whereas calcium may contribute to compensatory signaling and membrane stabilization under stress conditions. Species-specific patterns revealed distinct Cd-handling strategies, with cress and broccoli exhibiting high sensitivity, watercress showing partial physiological buffering, and white cabbage maintaining biomass despite efficient Cd transfer to edible tissues. Importantly, Cd-induced nutrient imbalance, together with enhanced Cd accumulation in aboveground tissues, represents a dual challenge by simultaneously reducing crop nutritional quality and increasing potential risks for human health. These findings emphasize the importance of species selection and management strategies aimed at limiting Cd uptake and translocation to safeguard both crop productivity and food safety.

Based on the integrated analysis, a conceptual response model can be proposed in which Cd uptake initiates ionic imbalance and oxidative stress signaling, triggering coordinated physiological adjustments, including pigment destabilization, growth inhibition, and species-dependent modulation of Cd partitioning between roots and shoots. These processes ultimately determine both tolerance capacity and potential food safety risk.

When growth responses and Cd accumulation patterns are evaluated together, clear species-dependent differences emerge in both tolerance and metal partitioning behavior; however, these responses do not always follow a consistent pattern. Importantly, the observed dissociation between growth performance and Cd accumulation highlights that physiological tolerance does not necessarily equate to food safety, particularly in crops that maintain biomass production while accumulating high Cd concentrations in edible tissues. This finding underscores the importance of integrating physiological and toxicological assessments when evaluating crop suitability in Cd-contaminated soils.

## 4. Materials and Methods

### 4.1. Experimental Design, Plant Material, and Growth Conditions

The experiment was conducted as a factorial pot experiment with two factors: Cd levels and *Brassicaceae* crop species. Five Cd levels (0, 5, 10, 20, and 50 mg kg^−1^ soil), supplied as cadmium chloride (CdCl_2_·H_2_O), and four *Brassicaceae* crops were evaluated. The experiment was arranged in a completely randomized design, with three biological replicates (pots) per treatment. Each experimental unit consisted of one pot, and treatments were randomly distributed within the greenhouse to minimize positional effects.

Seeds of four crops, cress (*Lepidium sativum*), watercress (*Eruca vesicaria*), broccoli (*Brassica oleracea* var. *italica*), and white cabbage (*Brassica oleracea* var. *capitata*), were sown in 15 cm-diameter plastic pots filled with 4 kg of soil. After seedling emergence, plants were thinned to maintain 20 uniform plants per pot for *Lepidium sativum* and *Eruca vesicaria* and 5 uniform plants per pot for *Brassica* species to ensure comparable plant density among species. The physicochemical properties of the experimental soil are presented in Table 4.

Basal fertilization was applied uniformly to all pots at rates of 150 mg kg^−1^ nitrogen (N), 75 mg kg^−1^ phosphorus (P), and 150 mg kg^−1^ potassium (K) using ammonium nitrate (NH_4_NO_3_), mono-potassium phosphate (KH_2_PO_4_), and potassium sulfate (K_2_SO_4_), respectively. Cd treatments were applied once prior to sowing using cadmium chloride monohydrate (CdCl_2_·H_2_O) as the Cd source. Target concentrations (0, 5, 10, 20, and 50 mg Cd kg^−1^) were calculated from elemental Cd, and the corresponding CdCl_2_·H_2_O mass was determined from the molecular weight to achieve the desired Cd levels. The CdCl_2_·H_2_O was fully dissolved in distilled water, gently heated to enhance solubility, and visually inspected to confirm complete dissolution and absence of precipitation before application. The resulting solution was uniformly incorporated into the soil, and treated soils were allowed to equilibrate for 24 h before sowing to facilitate stabilization of Cd–soil interactions and ensure homogeneous distribution. Plants were grown under controlled greenhouse conditions, and plant material within each pot was averaged to obtain one representative value per biological replicate in subsequent analyses.

### 4.2. Greenhouse Conditions and Plant Maintenance

The experiment was conducted during the 2024 summer season in the experimental greenhouses of the Faculty of Agriculture, Kocaeli University, Türkiye (40°40′47″ N, 30°01′37″ E). The study area is located within the Marmara transitional climatic zone, characterized by a humid temperate climate with moderate seasonal variability. Greenhouse conditions were maintained at an average daytime temperature of 27 °C and a nighttime temperature of 17 °C, with relative humidity ranging from 48% to 76%. Although environmental parameters were controlled inside the greenhouse, providing a regional climatic context improves reproducibility and facilitates comparison with similar studies conducted under different ecological conditions.

### 4.3. Cadmium Application and Soil Preparation

Cd treatments were prepared as aqueous solutions of CdCl_2_ and incorporated into the soil by spraying before seed sowing. Fertilizer solutions were applied simultaneously with Cd treatments to ensure homogeneous nutrient availability across all pots. After application, soils were thoroughly mixed by repeated manual turning and blending to achieve a uniform distribution of Cd and nutrients. The amended soils were allowed to equilibrate for 24 h before sowing to stabilize metal–soil interactions. This equilibration period was implemented to facilitate stabilization of Cd–soil interactions, allowing adsorption processes and ionic distribution within the soil matrix to reach equilibrium and thereby improving treatment consistency and reproducibility.

Cd treatments were applied once at the beginning of the experiment before sowing using cadmium chloride monohydrate (CdCl_2_·H_2_O), and no additional Cd applications were performed during the experimental period. This single-dose approach was selected to simulate a stable soil contamination scenario rather than repeated exposure conditions. To prevent potential Cd loss through leaching and maintain stable Cd concentrations in the soil, plastic liners were placed inside each pot before filling with soil to ensure water impermeability. Soil water-holding capacity (field capacity) was determined before the experiment, and irrigation was managed using a gravimetric approach. Pots were regularly weighed, and water lost through plant uptake and surface evaporation was replenished with distilled water to maintain soil moisture close to field capacity. This approach minimized changes in soil solution volume and prevented Cd loss from the soil system, thereby helping maintain the intended Cd exposure levels throughout the experimental period. These refinements ensured improved reproducibility, consistency, and clarity of the experimental procedures, particularly with respect to Cd application, irrigation control, and biochemical measurements.

### 4.4. Harvest and Sample Preparation

Plants were grown from sowing until harvest under controlled greenhouse conditions, and physiological and biochemical measurements were performed at the final harvest stage to assess cumulative responses to Cd exposure. After six weeks of Cd exposure, plants were harvested.

Shoots were cut at the soil surface, washed with running tap water, and rinsed three times with deionized water to remove adhering soil particles. Roots were carefully separated from the soil and immersed in an aerated 0.5 mM CaCl_2_ solution for 15 min to desorb loosely bound apoplastic ions, followed by thorough rinsing with deionized water.

Fresh plant tissues designated for biochemical analyses were processed immediately after harvest to avoid metabolic degradation.

Shoot and root samples intended for elemental and Cd analyses were oven-dried at 65 °C for 48 h until constant weight and weighed to determine dry biomass. Dried samples were ground to a fine powder using a laboratory mill for subsequent mineral nutrient and Cd concentration analyses.

### 4.5. Photosynthetic Pigment Analysis

Photosynthetic pigments were determined in the youngest fully expanded leaves sampled just before harvest. Fresh leaf tissue (200 mg) was homogenized in 10 mL of 90% (*v*/*v*) acetone and filtered through Whatman No. 42 filter paper. Absorbance was measured at 645, 663, 652, and 470 nm using a UV–visible spectrophotometer (Shimadzu UV-1201, Kyoto, Japan). Chlorophyll (Chl) *a*, Chl *b*, total Chl, and carotenoid (Car) contents were calculated according to Lichtenthaler [50].

### 4.6. Cadmium Determination and Accumulation Indices

For Cd determination, 0.5 g of oven-dried shoot or root material was subjected to dry ashing at 500 °C for 6 h in a muffle furnace. The resulting ash was dissolved in 5 mL of 0.1 M HCl and filtered before analysis. Cd concentrations were quantified using inductively coupled plasma optical emission spectrometry (ICP-OES; Perkin Elmer Optima 2100 DV, Waltham, MA, USA) following the procedure described by Miller [51].

Cd distribution, dry matter content (DMC), bio-concentration factor (BCF), translocation factor (TF), and total accumulation rate (TAR) were calculated using standard equations described by Ait Ali et al. [18] and Cikili et al. [19].

Cd distribution (%) = [(Cd)_shoot or root_/Cd_shoot_ + Cd_root_] × 100 (1)

DMC (%) = [(DW)_shoot, or root_/(FW)_shoot or root_] × 100 (2)

TF (%) = [Cd_shoot_/Cd_root_] × 100 (3)

BCF = (Cd)_shoot, or root_/(total Cd)_soil_ (4)

TAR of Cd (μg g^−1^ DW day^−1^) = [(Cd_shoot_ × DW_shoot_) + (Cd_root_ × DW_root_)]/growth day × (DW_shoot_ + DW_root_) (5)

where [total Cd]_soil_ = present Cd concentration in experimental soil + added Cd concentration for every Cd level

### 4.7. Oxidative Stress Parameters

Oxidative stress parameters were determined using fresh leaf tissues collected from the youngest fully expanded leaves immediately before harvest. All biochemical analyses were performed using 0.25 g of fresh plant material homogenized in appropriate extraction buffers under cold conditions to prevent biochemical degradation.

Non-enzymatic oxidative stress indicators, including hydrogen peroxide (H_2_O_2_), malondialdehyde (MDA), proline content, and membrane permeability (MP), were quantified using established spectrophotometric methods to evaluate Cd-induced oxidative responses in photo-synthetically active tissues. Antioxidant enzyme activities were not included in the present study, as the focus was primarily on non-enzymatic oxidative stress indicators.

H_2_O_2_ content was measured following the method of Mukherjee and Choudhuri [52], with absorbance recorded at 390 nm. Lipid peroxidation was evaluated by determining MDA content using the thiobarbituric acid (TBA) method described by Hodges et al. [53], with absorbance measured at 532 nm and corrected at 600 nm. Proline content was determined using the ninhydrin-based colorimetric assay of Bates et al. [54], with absorbance recorded at 520 nm.

Membrane permeability (MP) was assessed by measuring electrolyte leakage following Yan et al. [55]. Electrical conductivity was measured before and after boiling, and MP was calculated using the following equation:

MP (EC, %) = (C1/C2) × 100(6)

where C1 and C2 are the electrolyte conductivities measured before and after boiling, respectively.

### 4.8. Statistical Analysis

The experiment was arranged as a factorial design (Cd level × species) in a completely randomized design with three replicates (pots) per treatment. Each pot was considered the experimental unit. For each parameter, plant material within a pot was averaged to obtain a single value per experimental unit, thereby avoiding pseudo-replication.

Data normality was verified using the Shapiro–Wilk test prior to statistical analyses. Data were analyzed using two-way analysis of variance (ANOVA) with Cd level and species as fixed factors, including their interaction (Cd × species), using MINITAB software (Version 16; Minitab Inc., State College, PA, USA).

Pearson’s correlation coefficients (r) were calculated to evaluate relationships among physiological and biochemical parameters in shoot and root tissues, and correlation matrices were generated using XLSTAT 2023. Mean comparisons were performed using Duncan’s Multiple Range Test (DMRT) at a significance level of α = 0.05. Statistical significance was defined as *p* < 0.05 (*), *p* < 0.01 (**), *p* < 0.001 (***), and ns (not significant).

## 5. Conclusions

This study demonstrates that increasing Cd availability significantly disrupts growth performance, physiological processes, nutrient balance, and metal partitioning in Brassicaceae crops, with clear species-dependent differences in tolerance and Cd management strategies. Cd exposure resulted in marked growth inhibition, altered biomass allocation, impaired photosynthetic pigment stability, and enhanced oxidative stress responses, confirming the multifaceted impact of Cd toxicity on plant metabolism.

Substantial Cd accumulation occurred in both root and shoot tissues across all species, with roots serving as the primary Cd sink at lower Cd levels but progressively losing their retention capacity under higher Cd exposure. This shift led to increased root-to-shoot translocation, indicating a critical transition in Cd partitioning behavior under severe stress conditions.

Cd stress also induced significant imbalances in essential mineral nutrients, particularly K, Ca, and Zn, suggesting strong competitive interactions and disruption of nutrient uptake and transport processes. These nutrient imbalances likely contributed to the observed physiological impairments and further exacerbated Cd toxicity effects.

Importantly, the results revealed a clear dissociation between physiological tolerance and Cd accumulation, indicating that crops maintaining relatively stable growth under Cd stress may still accumulate high Cd levels in edible tissues, thereby posing potential food safety risks.

Overall, the integration of physiological responses, Cd partitioning dynamics, and nutrient interactions provides a comprehensive framework for evaluating crop performance under Cd-contaminated conditions. These findings highlight the importance of adopting integrated assessment approaches that consider both agronomic performance and food safety when selecting crops for cultivation in Cd-affected soils.

## Figures and Tables

**Table 1:** Effects of cadmium on concentration, distribution, and uptake of Cd in the shoots and roots of *Brassica* crops.

Applied Cd Levels (mg kg^−1^)	Dry Weights (DWs) (g pot^−1^)		Dry Matter Content (DMC) (%)		Cd Distribution (%)	
	Shoot	Root	Shoot	Root	Shoot	Root
Cress						
0	5.94 ± 0.33 a	2.07 ± 0.17 a	16.27 ± 0.50 a	11.84 ± 0.27 ab	11.67 ± 2.1 c	88.33 ± 2.1 a
5	5.03 ± 0.13 b	1.77 ± 0.18 a	15.10 ± 0.72 a	12.42 ± 2.50 ab	53.18 ± 1.6 a	46.82 ± 1.6 c
10	4.88 ± 0.34 b	1.76 ± 0.15 a	14.68 ± 0.46 a	9.65 ± 0.79 b	51.7 ± 1.7 a	48.22 ± 1.7 c
20	4.36 ± 0.46 b	1.22 ± 0.27 b	11.18 ± 1.22 b	10.96 ± 1.08 ab	34.61 ± 1.6 b	65.39 ± 1.6 b
50	2.87 ± 0.10 c	0.53 ± 0.05 c	7.79 ± 0.05 c	5.05 ± 1.20 c	14.02 ± 0.6 c	85.98 ± 0.5 a
Watercress						
0	6.92 ± 0.24 a	1.44 ± 0.25 a	14.93 ± 0.03 a	12.50 ± 3.00 a	14.29 ± 2.8 a–c	85.71 ± 2.8 ab
5	6.63 ± 0.22 a	1.24 ± 0.06 a	13.16 ± 0.33 a	11.12 ± 0.50 a	16.55 ± 0.4 a	83.45 ± 0.4 b
10	6.75 ± 0.31 a	1.25 ± 0.20 a	14.05 ± 0.52 ab	9.30 ± 1.73 ab	16.17 ± 0.3 ab	83.83 ± 0.3 b
20	5.73 ± 0.30 b	1.19 ± 0.13 a	11.95 ± 0.32 bc	9.02 ± 0.53 ab	12.81 ± 0.2 bc	87.19 ± 0.2 a
50	4.32 ± 0.14 c	1.03 ± 0.14 a	10.32 ± 1.10 c	6.00 ± 0.36 b	11.55 ± 0.4 c	88.45 ± 0.4 a
Broccoli						
0	2.95 ± 0.26 a	0.48 ± 0.23 a	9.86 ± 1.29 a	7.13 ± 4.92 a	1.88 ± 0.1 b	98.12 ± 0.1 a
5	2.56 ± 0.16 ab	0.33 ± 0.05 a	8.52 ± 0.51 ab	2.36 ± 0.34 b	2.83 ± 0.2 b	97.17 ± 0.2 a
10	2.19 ± 0.05 b	0.24 ± 0.02 a	5.83 ± 0.57 c	2.31 ± 0.25 ab	3.35 ± 0.3 b	96.65 ± 0.4 a
20	2.11 ± 0.07 b	0.22 ± 0.04 a	6.14 ± 0.27 c	1.81 ± 0.33 b	3.92 ± 0.1 b	96.08 ± 0.1 a
50	0.99 ± 0.15 c	0.13 ± 0.07 b	7.37 ± 0.32 bc	2.32 ± 0.15 b	12.32 ± 0.5 a	87.68 ± 0.5 b
White Cabbage						
0	6.06 ± 0.21 a	1.07 ± 0.07 a	10.36 ± 0.62 a	2.21 ± 0.24 a	3.16 ± 0.5 d	96.84 ± 0.5 a
5	6.11 ± 0.13 a	1.02 ± 0.08 a	11.07 ± 1.01 a	2.18 ± 0.22 a	18.33 ± 1.3 a	81.67 ± 1.3 d
10	5.94 ± 0.06 a	0.99 ± 0.17 a	10.11 ± 0.52 a	1.77 ± 0.20 a	12.46 ± 0.5 bc	87.54 ± 0.5 bc
20	5.64 ± 0.32 a	0.95 ± 0.08 a	10.36 ± 0.74 a	2.50 ± 0.25 a	10.60 ± 0.9 b	89.40 ± 0.9 b
50	3.54 ± 0.19 b	0.52 ± 0.04 b	7.53 ± 0.58 b	3.29 ± 1.17 a	14.69 ± 1.0 c	85.31 ± 1.0 c
LSD (%5)	0.669	0.332	1.933	4.442	3.224	3.226
ANOVA: *F* values						
Genera (G)	269.81 ***	69.38 ***	75.03 ***	34.06 ***	569.35 ***	569.62 ***
Cd	71.54 ***	15.04 ***	28.41 ***	4.10 ***	114.71 ***	114.78 ***
G × Cd	1.59 *	2.93 ***	5.13 ***	1.39 ***	79.25 ***	79.30 ***

Values are means ± SE (*n* = 3). Different letters within the same column for each variety indicate significant differences according to DMRT (*α* < 0.05); * and *** indicate significance at *p* < 0.05 and *p* < 0.001.

**Table 2:** Effects of cadmium levels on Cd concentrations, BCF, TF, and TAR of Cd in the shoots and roots of *Brassica* crops.

Added Cd to Soils (mg kg^−1^)	Cd Concentrations (mg kg^−1^)		BCF of Cd		TF of Cd (%)	TAR of Cd (μg g^−1^ DW day^−1^)
	Shoot	Root	Shoot	Root		
Cress						
0	0.80 ± 0.12 e	6.13 ± 0.37 c	20.0 ± 2.89 a	153.3 ± 9.29 a	13.33 ± 2.70 c	1.83 ± 0.23 c
5	29.60 ± 0.92 d	26.07 ± 1.07 c	5.9 ± 0.18 b	5.2 ± 0.20 b	114.10 ± 7.25 a	17.40 ± 0.57 bc
10	55.07.85 c	51.40 ± 2.66 c	5.5 ± 0.09 b	5.1 ± 0.24 b	107.83 ± 6.98 a	31.67 ± 4.83 ab
20	84.07 ± 1.62 b	159.90 ± 11.9 b	4.2 ± 0.06 b	8.0 ± 0.55 b	53.13 ± 3.84 b	42.03 ± 9.77 a
50	172.08 ± 3.67 a	1063.10 ± 49.7 a	3.4 ± 0.09 b	21.3 ± 0.99 b	16.30 ± 0.74 c	47.87 ± 6.10 a
Watercress						
0	1.20 ± 0.20 e	7.33 ± 0.59 e	30.0 ± 5.00 a	183.3 ± 14.8 a	16.93 ± 3.77 a	1.80 ± 0.20 d
5	25.33 ± 0.24 d	127.93 ± 3.62 d	5.0 ± 0.03 b	25.4 ± 0.71 b	19.83 ± 0.58 a	34.33 ± 1.17 c
10	63.93 ± 2.82 c	331.07 ± 7.18 c	6.4 ± 0.27 b	33.0 ± 0.72 b	19.30 ± 0.45 a	97.00 ± 8.06 b
20	85.20 ± 1.93 b	579.60 ± 5.14 b	4.2 ± 0.09 b	28.9 ± 0.26 b	14.70 ± 0.27 a	108.03 ± 9.80 b
50	195.20 ± 12.2 a	1500.01 ± 116 a	3.9 ± 0.25 b	30.0 ± 2.32 b	13.03 ± 0.49 a	166.03 ± 9.07 a
Broccoli						
0	0.93 ± 0.07 e	48.67 ± 1.03 e	23.3 ± 1.67 a	1217.2 ± 25.3 a	1.93 ± 0.12 b	0.37 ± 0.12 a
5	7.27 ± 0.66 d	249.03 ± 3.20 d	1.4 ± 0.12 b	49.4 ± 0.64 b	2.93 ± 0.22 b	3.93 ± 0.83 a
10	13.33 ± 1.10 c	386.10 ± 11.8 c	1.3 ± 0.12 b	38.4 ± 1.17 b	3.47 ± 0.35 b	3.97 ± 0.23 a
20	23.80 ± 1.10 b	582.60 ± 12.6 b	1.2 ± 0.06 b	29.1 ± 0.64 b	4.41 ± 0.12 b	7.10 ± 0.51 a
50	172.27 ± 6.45 a	1229.30 ± 68.5 ba	3.4 ± 0.12 b	4.6 ± 1.39 c	14.07 ± 0.60 a	5.10 ± 1.18 a
White Cabbage						
0	0.60 ± 0.12 e	18.20 ± 0.61 d	15.0 ± 2.89 a	455.0 ± 15.3 a	3.30 ± 0.55 c	2.13 ± 0.07 d
5	26.80 ± 2.60 d	118.87 ± 1.64 d	5.3 ± 0.52 b	23.6 ± 0.32 b	22.50 ± 2.04 a	26.60 ± 1.85 c
10	35.13 ± 1.12 c	247.50 ± 11.1 c	3.5 ± 0.12 b	24.6 ± 1.92 b	14.23 ± 0.64 b	55.57 ± 9.89 b
20	63.20 ± 2.37 b	538.50 ± 36.8 b	3.2 ± 0.25 b	26.9 ± 3.16 b	11.90 ± 1.11 b	75.30 ± 7.54 a
50	251.60 ± 16.8 a	1462.90 ± 42.7 a	5.0 ± 0.61 b	29.2 ± 1.50 b	17.27 ± 1.35 b	88.60 ± 4.21 a
LSD (%5)	14.76	99.93	4.294	22.08	7.758	17.13
ANOVA: *F* values						
Genera (G)	444.70 ***	385.47 ***	6.59 **	929.88 ***	422.57 ***	150.19 ***
Cd	1437.73 ***	1154.37 ***	117.24 ***	3050.06 ***	97.07 ***	100.04 ***
G × Cd	87.07 ***	61.68 ***	4.04 ***	798.98 ***	77.03 ***	20.23 ***

Values are means ± SE (*n* = 3). Different letters within the same column for each variety indicate significant differences according to DMRT (*α* < 0.05); ** and *** indicate significance at *p* < 0.01 and *p* < 0.001, respectively.

**Table 3:** Effects of cadmium levels on some ion concentrations in the shoots and roots of *Brassica* crops.

Added Cd (mg kg^−1^)	Potassium (K) (mg kg^−1^)		Calcium (Ca) (mg kg^−1^)		Zinc (Zn) (mg kg^−1^)	
	Shoot	Root	Shoot	Root	Shoot	Root
Cress						
0	39.38 ± 0.97 c	16.84 ± 0.34 b	0.83 ± 0.03 b	0.26 ± 0.01 a	40.60 ± 1.15 c	34.47 ± 0.87 c
5	40.71 ± 0.98 c	11.96 ± 0.16 c	0.81 ± 0.03 b	0.26 ± 0.01 a	40.93 ± 1.28 c	38.07 ± 0.64 c
10	41.73 ± 1.82 c	15.19 ± 0.22 b	0.75 ± 0.01 b	0.24 ± 0.01 a	47.60 ± 0.81 b	39.67 ± 6.07 c
20	60.56 ± 2.25 b	16.47 ± 0.80 b	1.05 ± 0.01 b	0.28 ± 0.01 a	58.67 ± 1.52 a	81.20 ± 1.03 b
50	71.68 ± 3.92 a	19.42 ± 0.51 a	5.00 ± 0.50 a	0.48 ± 0.01 a	42.47 ± 1.07 c	111.93 ± 7.13 a
Watercress						
0	36.43 ± 0.94 ab	12.25 ± 0.49 c	3.61 ± 0.05 b	0.39 ± 0.02 a	21.87 ± 0.88 c	41.20 ± 1.50 d
5	38.67 ± 0.82 ab	16.15 ± 0.16 b	4.02 ± 0.16 b	0.32 ± 0.02 a	23.80 ± 0.72 c	71.00 ± 2.57 c
10	35.03 ± 1.7 3 b	18.07 ± 1.65 ab	4.33 ± 0.16 b	0.41 ± 0.04 a	27.20 ± 1.14 b	73.80 ± 1.93 c
20	39.43 ± 0.50 ab	19.33 ± 1.04 a	4.87 ± 0.06 ab	0.47 ± 0.04 a	27.67 ± 0.77 b	84.40 ± 4.10 b
50	41.32 ± 0.48 a	15.93 ± 0.29 b	6.12 ± 0.05 a	0.71 ± 0.04 a	35.93 ± 1.21 a	141.53 ± 6.76 a
Broccoli						
0	59.76 ± 3.30 c	35.24 ± 0.26 a	12.83 ± 0.58 ab	4.25 ± 0.54 b	16.73 ± 0.57 c	47.17 ± 1.34 b
5	70.48 ± 1.25 a	31.14 ± 1.29 b	11.42 ± 0.90 b	7.28 ± 0.63 a	18.47 ± 0.41 c	48.17 ± 0.77 b
10	65.16 ± 0.62 b	20.37 ± 1.02 c	11.45 ± 0.30 b	6.77 ± 0.34 a	13.53 ± 0.24 d	33.60 ± 1.42 c
20	56.68 ± 1.39 c	30.12 ± 0.67 b	12.45 ± 0.55 ab	1.80 ± 0.15 c	32.13 ± 1.16 b	50.47 ± 2.15 ab
50	46.90 ± 1.23 d	11.67 ± 0.44 d	13.68 ± 1.64 a	2.14 ± 0.07 c	41.73 ± 0.93 a	58.33 ± 4.41 a
White Cabbage						
0	54.05 ± 0.65 b	22.39 ± 0.96 b	5.45 ± 0.18 b	0.63 ± 0.03 b	28.40 ± 0.53 a	29.27 ± 1.16 b
5	56.08 ± 1.92 ab	21.47 ± 0.74 b	5.35 ± 0.08 b	0.47 ± 0.04 b	26.27 ± 0.77 b	34.53 ± 1.74 b
10	56.61 ± 0.15 ab	21.39 ± 0.17 b	5.06 ± 0.19 b	0.77 ± 0.11 b	26.80 ± 1.44 b	33.20 ± 1.51 b
20	55.40 ± 0.87 ab	27.77 ± 0.83 a	5.13 ± 0.05 b	0.84 ± 0.03 b	27.93 ± 1.67 b	52.53 ± 0.82 a
50	59.96 ± 1.06 a	15.73 ± 0.63 c	7.21 ± 0.16 a	2.89 ± 0.19 a	38.93 ± 1.95 a	50.80 ± 3.70 a
LSD (%5)	4.65	2.15	1.38	0.61	3.08	9.35
ANOVA: *F* values						
Genera (G)	170.53 ***	193.02 ***	439.10 ***	419.39 ***	402.18 ***	56.66 ***
Cd	12.98 ***	61.72 ***	20.78 ***	23.32 ***	119.57 ***	876.14 ***
G × Cd	33.72 ***	53.04 ***	1.82 ^ns^	47.69 ***	33.80 ***	9.23 ***

Values are means of three replicates (mean ± SE, *n* = 3). This indicates that *DMRT%*5 (genera × Cd) was significantly different according to Duncan’s multiple range test (*p* < 0.05). Different letters within the same column for each variety indicate significant differences according to DMRT (*α* < 0.05); ANOVA shows a significant difference at *** *p* < 0.001 and ns: non-significant.

**Table 4:** Some characteristics of experimental soil.

Properties/Method	Amount	Properties/Method	Amount
pH/1:2.5 soil/water extraction	7.34	Available-Mg (mg kg^−1^)/NH_4_OAc-extractable	124
EC (μS cm^−1^)/saturation extraction	508	Available-Na (mg kg^−1^)/NH_4_OAc-extractable	64
CaCO_3_ (g kg^−1^)/calsimeter	17.29	Available-Fe (mg kg^−1^)/DTPA-extractable	24.28
Organic-C (g kg^−1^)/Walkley–Black	6.25	Available-Mn (mg kg^−1^)/DTPA-extractable	65.27
Total-N (g kg^−1^)/Kjeldahl	0.86	Available-Zn (mg kg^−1^)/DTPA-extractable	2.09
Available-P (mg kg^−1^)/NaHCO_3_-available	12.43	Available-Cu (mg kg^−1^)/DTPA-extractable	1.17
Available-K (mg kg^−1^)/NH_4_OAc-extractable	100	Available-B (mg kg^−1^)/hot water-extractable	1.64
Available-Ca (mg kg^−1^)/NH_4_OAc-extractable	2151	Cd (mg kg^−1^)/DTPA-extractable	0.04
Texture/Hydrometer	Loamy clay (35.8% sand, 21.7% clay)		

## Data Availability

All raw data supporting the conclusion in this article will be available from the author upon request.
